# Identification of potential tissue-specific cancer biomarkers and development of cancer versus normal genomic classifiers

**DOI:** 10.18632/oncotarget.21127

**Published:** 2017-09-21

**Authors:** Akram Mohammed, Greyson Biegert, Jiri Adamec, Tomáš Helikar

**Affiliations:** ^1^ Department of Biochemistry, University of Nebraska-Lincoln, Lincoln, Nebraska, USA

**Keywords:** cancer classification, biomarker identification, microarray gene expression, machine learning, cancer biomarker

## Abstract

Machine learning techniques for cancer prediction and biomarker discovery can hasten cancer detection and significantly improve prognosis. Recent “OMICS” studies which include a variety of cancer and normal tissue samples along with machine learning approaches have the potential to further accelerate such discovery. To demonstrate this potential, 2,175 gene expression samples from nine tissue types were obtained to identify gene sets whose expression is characteristic of each cancer class. Using random forests classification and ten-fold cross-validation, we developed nine single-tissue classifiers, two multi-tissue cancer-versus-normal classifiers, and one multi-tissue normal classifier. Given a sample of a specified tissue type, the single-tissue models classified samples as cancer or normal with a testing accuracy between 85.29% and 100%. Given a sample of non-specific tissue type, the multi-tissue bi-class model classified the sample as cancer versus normal with a testing accuracy of 97.89%. Given a sample of non-specific tissue type, the multi-tissue multi-class model classified the sample as cancer versus normal and as a specific tissue type with a testing accuracy of 97.43%. Given a normal sample of any of the nine tissue types, the multi-tissue normal model classified the sample as a particular tissue type with a testing accuracy of 97.35%. The machine learning classifiers developed in this study identify potential cancer biomarkers with sensitivity and specificity that exceed those of existing biomarkers and pointed to pathways that are critical to tissue-specific tumor development. This study demonstrates the feasibility of predicting the tissue origin of carcinoma in the context of multiple cancer classes.

## INTRODUCTION

Cancer has been characterized as a heterogeneous disease that is categorized by many different types and subtypes. In the United States, cancer is the second leading cause of death. In 2016, over 1.6 million new cases of cancer were diagnosed and over 600,000 people died from this disease; the disease accounts for approximately 23% of all deaths in the US each year [1]. Successful treatment depends on the timely diagnosis, and the five-year survival rate significantly increases with early detection. Diagnosis typically begins with symptomology, is supported by imaging technology, and is confirmed histopathologically by biopsy. These methods, however, suffer from low sensitivity and high costs.

The identification of cancer-specific biomarkers is being evaluated as an alternative diagnostic and treatment option since it is minimally invasive and thus has the potential to lower the cost of diagnosis. Already, several biomarkers have been identified and used to some extent in diagnosis; however, they usually have low accuracy, selectivity, and specificity, and high false-positive, false-negative rates of diagnosis [2]. Therefore, improving the process and tools for the discovery of new biomarkers is essential for future improvement in cancer diagnostics and successful treatment.

While many strategies for discovering biomarkers exist, selecting useful biomarkers is a challenging task [3, 4]. Examples of these strategies include gene-expression profiling, mass-spectrometry-based proteomic profiling, protein arrays and secreted protein approach [5]. Genomic and proteomic technologies have increased the number of potential biomarkers under investigation [6]. Furthermore, analysis of a single biomarker or a combination of only a few is increasingly being replaced by multiparametric analysis of genes, RNA, or proteins [7–10]. Specifically, high-throughput techniques such as microarrays and several machine-learning methods have been developed to study cancer classification and discovery of potential biomarkers [6, 11–20].

Many conventional biomarkers were established via discriminant analysis, through the comparison of cancerous tissues with normal tissues [21] or identifying nuanced differences among cancer subtypes [22, 23]. Progress in cancer biomarker identification has come through the application of machine learning to the analysis of high-throughput data from microarrays [24–29]. However, challenges remain in the application of machine learning to analyze biomarker data due to small sample sizes, the sheer size, and complexity of each dataset, as well as the diversity of experimental design [30].

The biomarker identification strategy outlined in this paper involves selecting genes whose differential expression in building cell structure, maintaining homeostasis, or the progression of cancer is a discriminating factor [31–34]. To achieve this, we developed single-tissue and multi-tissue machine learning cancer-versus-normal-classifiers using gene expression data that were used to identify tissue-specific cancer biomarkers. These biomarkers were obtained from machine learning models using gene expression data obtained and normalized from 2,175 samples and span nine tissue types. A feature selection method was identified to select informative genes (predicted biomarkers) from preprocessed data. Machine-learning models were identified through the analysis of gene expression samples from human cancer and non-cancerous tissue types that accurately distinguish malignant tissue from normal tissue and different malignant tissue types from each other. Using functional characterization and pathway analysis, the known tissue-specific cancer-related pathways were validated, and novel cancer-related pathways and functional groups for each of the tissue-specific predicted biomarkers were identified. The diagnostic capacity of the biomarkers predicted by the methods in this study (and later assessed by comparing their sensitivity and specificity to the sensitivity and specificity of known biomarkers for all tissue types) showed significant improvements over existing biomarkers. The development of our cancer prediction models and identification of the potential biomarkers may facilitate accurate, unbiased cancer diagnosis and effective treatment, ultimately improving cancer prognoses. Furthermore, the gene-expression signatures discovered by this classification approach may lead to new clinical reagents for successful tumor diagnosis.

## RESULTS

### Identification of the best feature selection algorithm

**Figure 1:**
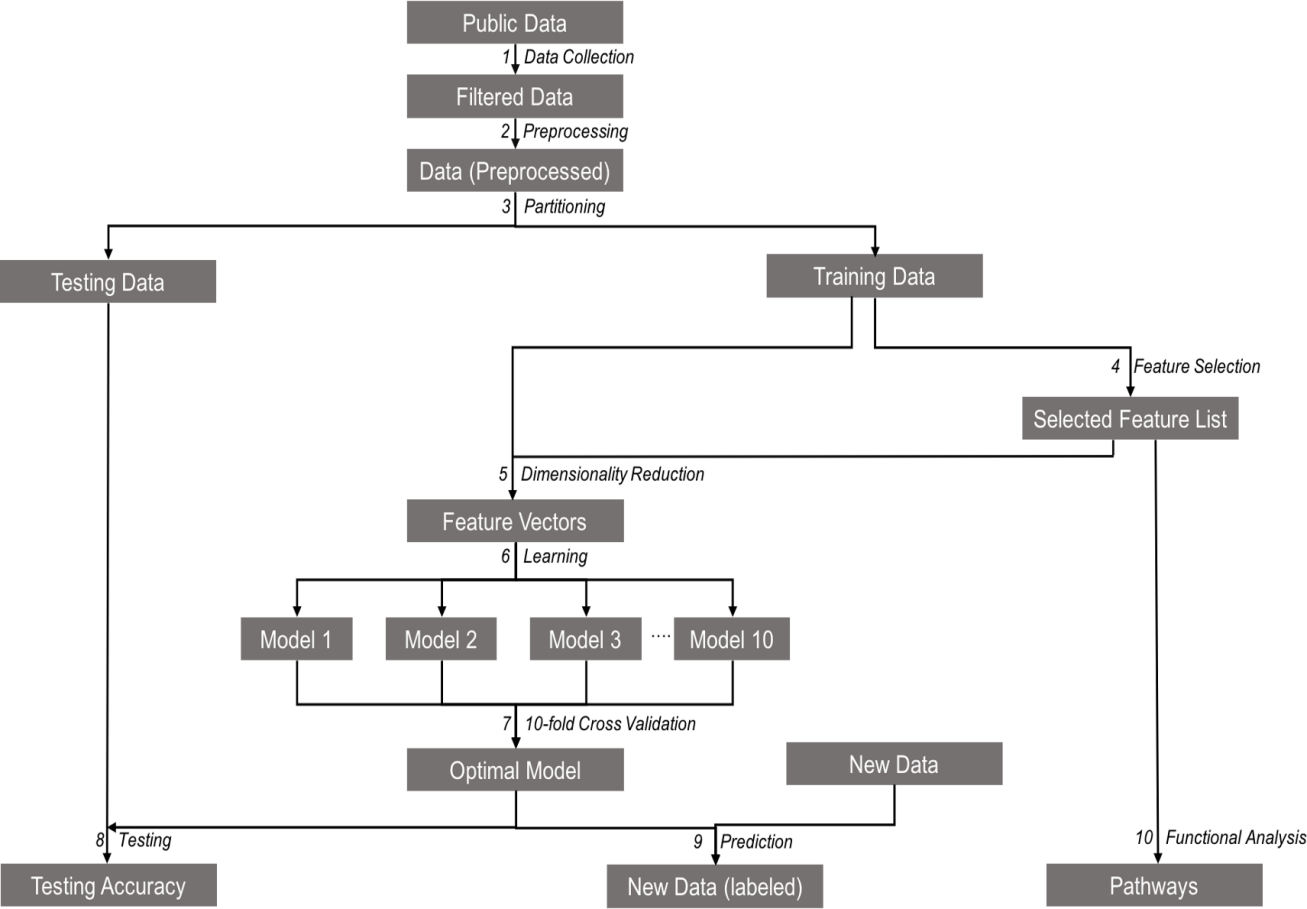
Schematic representation of the study workflow for each model (1) Microarray gene expression data for each tissue type relevant to the model were collected from the NCBI Gene Expression Omnibus (GEO) repository. (2) The data were then normalized, and background correction was performed on these data. (3) The preprocessed data were then partitioned into training and testing sets. (4) Feature selection was conducted on the training dataset to extract the list of important genes. (5) The list of selected genes was then mapped to the training data to generate the feature vectors using a process called Dimensionality Reduction. (6) Feature vectors were trained to create multiple models. (7) Ten-fold cross-validation was used to identify the optimal model. (8) The model performance was assessed by the testing its accuracy using the testing dataset. (9) The model was used to predict the class labels for the samples in the unknown dataset. (10) The functional analysis was performed using the selected genes to retrieve the pathways and functional groups.

Of all the combinations of feature selection algorithms and feature thresholds tested (Step 4 in Figure 1), the Filtered Attribute Evaluator with Ranker method (FAER) used with a feature threshold of the top 1% genes performed the best (Supplementary Figure 1 shows the workflow for identification of the best feature selection algorithm and Supplementary File 1 provide the performance details of the feature selection algorithms; see Methods for the list of feature selection algorithms and feature thresholds). As such, FAER with a feature threshold of 1% was used for feature selection throughout this study.

### Predictive power of the models

#### Single-tissue models

**Figure 2:**
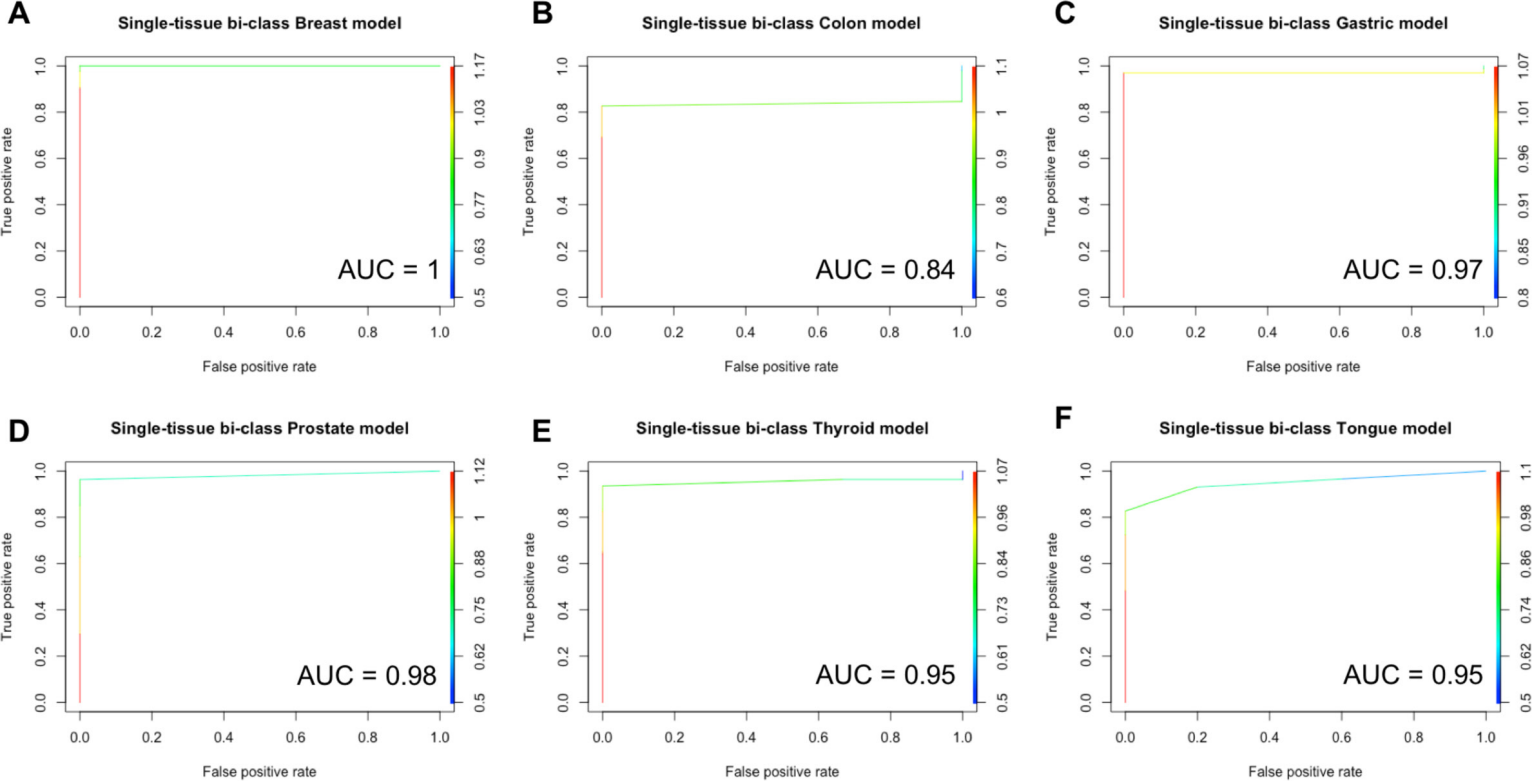
ROC for single-tissue specific models The area under the ROC curves is shown for each model. (**A**) Breast, (**B**) Colon, (**C**) Gastric, (**D**) Prostate, (**E**) Thyroid, and (**F**) Tongue. The ROC for the Blood, Head & Neck and Lung models are not shown due to the due to their AUC = 1.

Given a sample of a specific tissue type, single-tissue models accurately classify the sample as cancer or normal. Each single-tissue model more accurately classified samples from the same tissue type (same-tissue) than it classified samples from other tissue types (across-tissues). The area under the ROC (receiver operating characteristics) curve for tissue-specific models ranged from 0.84 (Colon model) to 1 and is shown in Figure 2. Same-tissue testing accuracies ranged from 85.29% (Tongue Model) to 100% (Blood, Head and Neck and Lung Models). Across-tissues test accuracies ranged from 33.46% (Lung Model) to 88.68% (Gastric Model). (More details can be found in Supplementary Figure 2 and Supplementary Table 1).

Among the two classifiers, the random-forests classifier performed better than the Support Vector Machine classifier for each model except the Tongue model (the Random Forests classifier yielded an 85.29% same-tissue testing accuracy compared to 94.11% by the Support Vector Machine classifier). The Random Forests classifier outperformed the Support Vector Machine classifier in the across-tissues testing accuracies for each model. Supplementary Figure 3A and 3B show the same-tissue and across-tissues accuracies respectively. (To see differences between the performances of these classifiers, see Supplementary Table 2 and Supplementary Table 3). As a result, the models were constructed with Random Forests for the duration of this study.

A list of 244 genes (predicted biomarkers) was identified for each tissue type (See Supplementary File 2 for complete list of biomarkers for each tissue type and Table 2 for the number of characterized and uncharacterized genes for each tissue type) is given in Supplementary File 3 whereas, the list of uncharacterized genes is provided in Table 3.

**Table 1:** Precision, recall and F1-Score for the multi-tissue bi-class model for training and testing data

	Training					Testing				
Class of Samples	# of Tumor Samples	# of Normal Samples	Precision (%)	Recall (%)	F1-Score	# of Tumor Samples	# of Normal Samples	Precision (%)	Recall (%)	F1-Score
Tumor	849	9	98.95	97.70	98.32	854	4	99.53	97.82	98.67
Normal	20	209	91.27	95.87	93.513	19	211	91.74	98.14	94.83

**Table 2:** Distribution of characterized and uncharacterized genes for each tissue type

Tissue	Predicted Biomarkers (Characterized Genes)	Predicted Biomarkers (Uncharacterized Genes)
Blood	170	74
Breast	239	5
Colon	240	4
Gastric	238	6
Head & Neck	243	1
Lung	224	20
Prostate	238	6
Thyroid	240	4
Tongue	237	7

**Table 3:** List of uncharacterized genes predicted as biomarkers from different tissue types

Potential Biomarker (Uncharacterized genes)	Tissue
CTC-265F19.1	Blood
CTC-360P9.3	Blood
CTC-378H22.2	Blood
CTC-384G19.1	Blood
CTC-400I9.1	Blood
CTC-428G20.6	Blood
CTC-436K13.5	Blood
CTC-459F4.3	Blood
CTC-462L7.1	Blood
CTC-471C19.1	Blood
CTC-471F3.6	Blood
CTC-471J1.2	Blood
CTC-527H23.4	Blood
CTC-550B14.7	Blood
CTD-2002J20.1	Blood
CTD-2008P7.1	Blood
CTD-2012K14.6	Blood
CTD-2021H9.3	Blood
CTD-2033C11.1	Blood
CTD-2035E11.5	Blood
CTD-2036P10.3	Blood
CTD-2076M15.1	Blood
CTD-2083E4.4	Blood
CTD-2083E4.7	Blood
CTD-2118P12.1	Blood
CTD-2130O13.1	Blood
CTD-2196E14.6	Blood
CTD-2199O4.3	Blood
CTD-2199O4.7	Blood
CTD-2251F13.1	Blood
CTD-2256P15.2	Blood
CTD-2269F5.1	Blood
CTD-2281E23.2	Blood
CTD-2284J15.1	Blood
CTD-2286N8.2	Blood
CTD-2287O16.5	Blood
CTD-2293H3.1	Blood
CTD-2302E22.4	Blood
CTD-2310F14.1	Blood
CTD-2311B13.7	Blood
CTD-2313J17.5	Blood
CTD-2314B22.3	Blood
CTD-2325A15.5	Blood
CTD-2366F13.2	Blood
CTD-2373J6.1	Blood
CTD-2377D24.6	Blood
CTD-2520I13.1	Blood
CTD-2534I21.8	Blood
CTD-2537I9.16	Blood
CTD-2537I9.5	Blood
CTD-2540F13.2	Blood
CTD-2541J13.1	Blood
CTD-2541M15.1	Blood
CTD-2542L18.1	Blood
CTD-2547L24.4	Blood
CTD-2553C6.1	Blood
CTD-2554C21.3	Blood
CTD-2555O16.4	Blood
CTD-2561B21.11	Blood
CTD-2587H24.10	Blood
CTD-2587M23.1	Blood
CTD-2611O12.6	Blood
CTD-2616J11.10	Blood
CTD-2619J13.13	Blood
CTD-2619J13.17	Blood
CTD-2639E6.4	Blood
CTD-2647L4.1	Blood
CTD-3028N15.1	Blood
CTD-3046C4.1	Blood
LOC730139	Blood
LOC731424	Blood
LOC80154	Blood
LOC90834	Blood
LQFBS-1	Blood
AX746733	Breast
RP11-114H24.6	Breast
RP11-255C15.3	Breast
RP11-348B17.1	Breast
RP11-403P17.4	Breast
LA16C-381G6.1	Colon
LOC100652770	Colon
RP11-295M18.6	Colon
RP11-38P22.2	Colon
GS1-103B18.1	Gastric
GS1-111G14.1	Gastric
GS1-18A18.2	Gastric
GS1-124K5.9	Gastric
GS1-164F24.1	Gastric
GS1-304P7.2	Gastric
FLJ11292	Head And Neck
RP11-69I8.2	Lung
RP3-406C18.2	Lung
RP4-710M16.1	Lung
AC007967.3	Lung
LOC613037	Lung
LOC100127886	Lung
RP1-217P22.2	Lung
AC009947.3	Lung
RP11-770J1.4	Lung
RP11-209A2.1	Lung
RP5-1184F4.5	Lung
MGC13053	Lung
RP3-391O22.2	Lung
LOC649330	Lung
RP3-406P24.1	Lung
RP13-258O15.1	Lung
RP5-1118D24.2	Lung
GS1-124K5.9	Lung
RP1-190J20.2	Lung
RP1-192P9.1	Lung
AC004941.5	Prostate
LOC100506119	Prostate
RP1-101G11.2	Prostate
RP11-297L17.2	Prostate
AX746823	Prostate
RP11-96K19.4	Prostate
RP6-24A23.7	Thyroid
LOC100506558	Thyroid
LOC101930400	Thyroid
LOC102725271	Thyroid
CTA-384D8.35	Tongue
RP11-353N14.2	Tongue
CTC-444N24.11	Tongue
RP11-539I5.1	Tongue
LOC101928615	Tongue
GS1-111G14.1	Tongue
RP11-250B2.3	Tongue

#### Multi-tissue bi-class model

**Figure 3:**
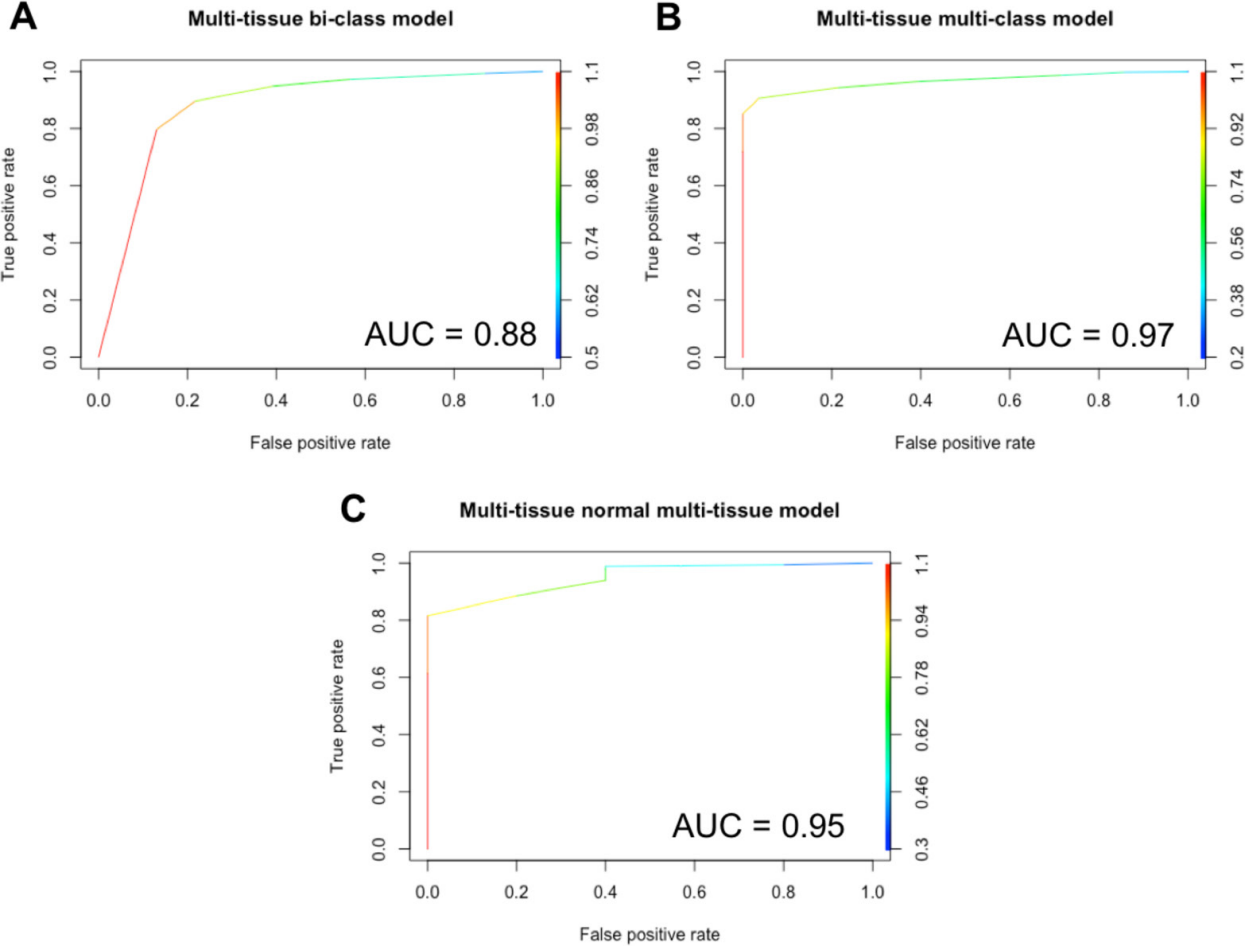
ROC for multi-tissue models The area under the ROC curves is shown for each model. (**A**) multi-tissue bi-class model, (**B**) multi-tissue multi-class model, (**C**) multi-tissue normal multi-class model.

Given a sample of any of nine tissue types, the multi-tissue bi-class model accurately classifies the sample as cancer or normal. The area under the ROC curve for the multi-tissue bi-class model is 0.88 and is shown in Figure 3A. The multi-tissue bi-class model achieved training and testing accuracies of 97.33% and 97.89%, respectively. The model was more accurate in predicting a Cancer sample (Precision and Recall of 98.95% and 97.70%, respectively) than a Normal sample (Precision and Recall of 91.27% and 95.87%, respectively). (See Table 1 for Precision, Recall, and F1- score measures for both training and testing datasets.)

#### Multi-tissue multi-class model

**Figure 4:**
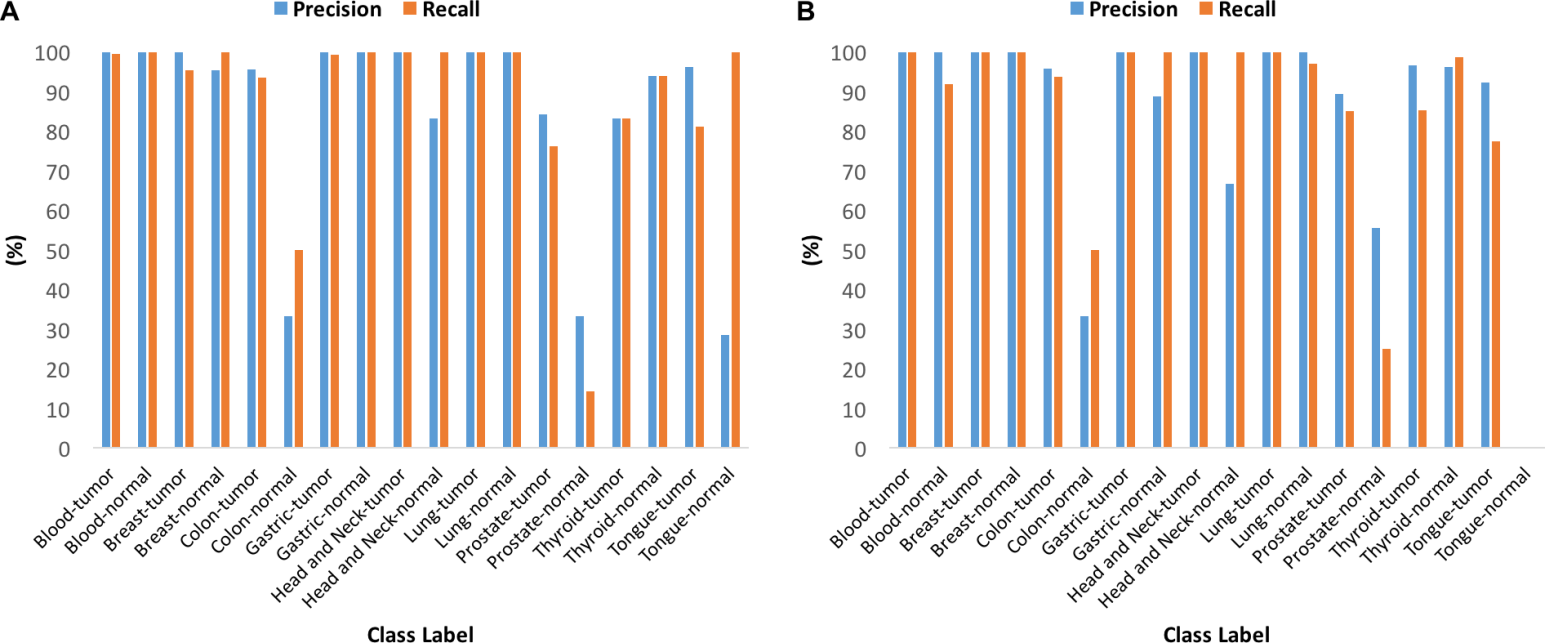
Performance of the multi-tissue multi-class models for each class (**A**) Precision and recall using the training dataset. (**B**) Precision and recall using the testing dataset.

Given a sample of any of the nine tissue types, the multi-tissue multi-class model accurately classifies the sample as cancer or normal, and as of a specific tissue type. The area under the ROC curve for the multi-tissue multi-class model is 0.97 and is shown in Figure 3B. The multi-tissue multi-class models achieved training and testing accuracies of 96.96% and 97.43%, respectively. The precision, recall, and F1- score for these models varied among classes (Figure 4). For the following classes, the model had 100% precision using the training dataset: blood-tumor, blood-normal, breast-tumor, gastric-tumor, gastric-normal, and head and neck-tumor. For the following classes, the model had 100% recall using the training dataset: blood-normal, gastric-normal, head-and neck-tumor, head-and-neck normal, lung-tumor, lung-normal, and tongue-normal. Out of all the classes, colon-normal (precision: 33.33%), prostate-normal (precision: 33.33%), and tongue-normal (precision: 28.57%) had the lowest precision using the training dataset (See Supplementary Table 4–6 for precision, recall, F1-score and confusion matrices).

#### Multi-tissue normal multi-class model

**Figure 5:**
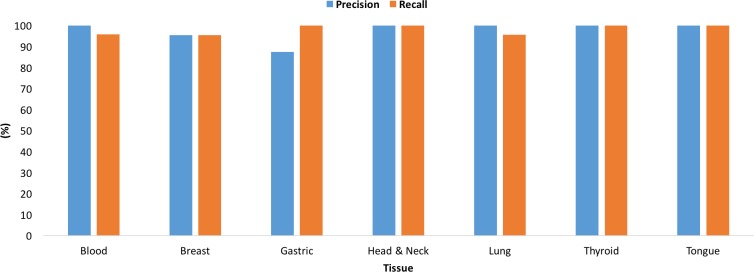
Performance of the multi-tissue normal multi-class model for each class Precision and Recall values are shown for each of the nine tissue types using the testing dataset.

Given a normal sample of any of the nine tissue types, the multi-tissue normal multi-class model accurately classifies the sample as of a particular tissue type. The area under the ROC curve for the multi-tissue normal multi-class model is 0.95 and is shown in Figure 3C. The multi-tissue normal multi-class models achieved training and testing accuracies of 97.88% and 97.35%, respectively. The models’ precision and recall for each normal class using the testing dataset ranged from 87.5% to 100%, and from 95.45% to 100%, respectively (See Figure 5, Supplementary Tables 7–9 for details).

### Functional analysis

#### Enrichment of cancer tissue-specific genes in metabolic and signaling pathways

**Figure 6:**
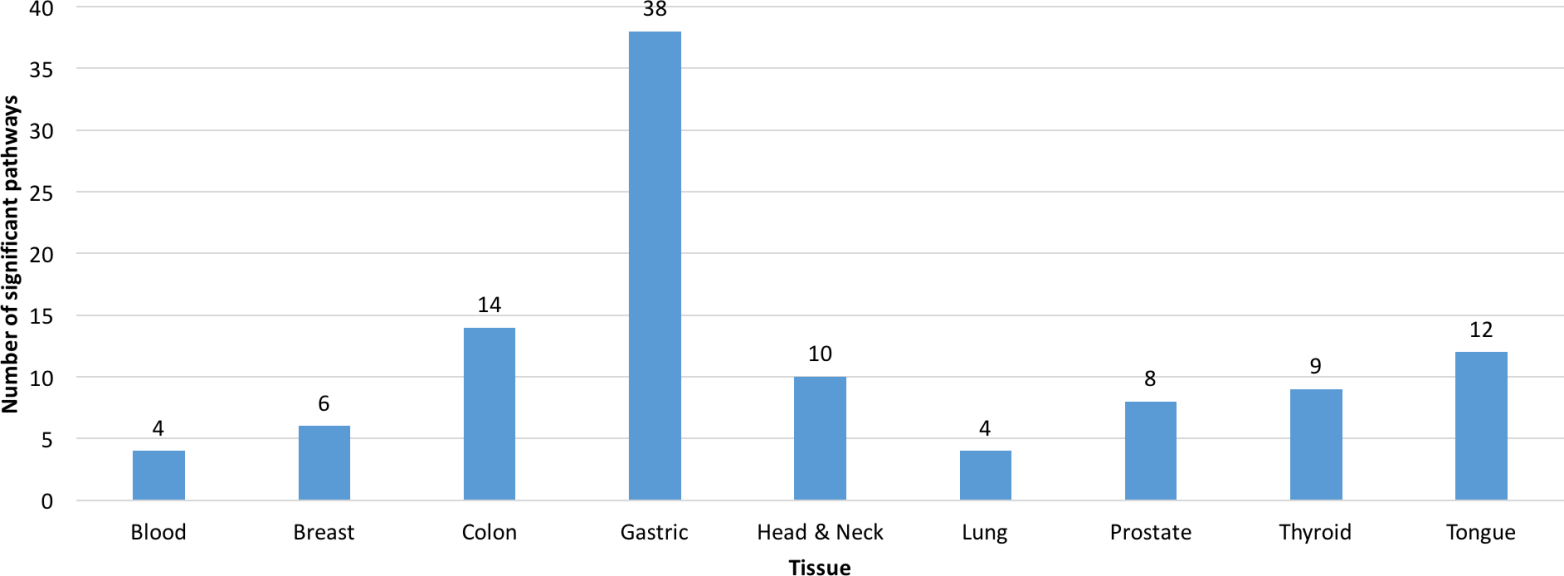
Number of significant pathways for the genes (predicted biomarkers) from each tissue type A pathway was significant if its *p*-value was less than or equal to 0.05 and it had a minimum of three tissue-specific genes.

**Figure 7:**
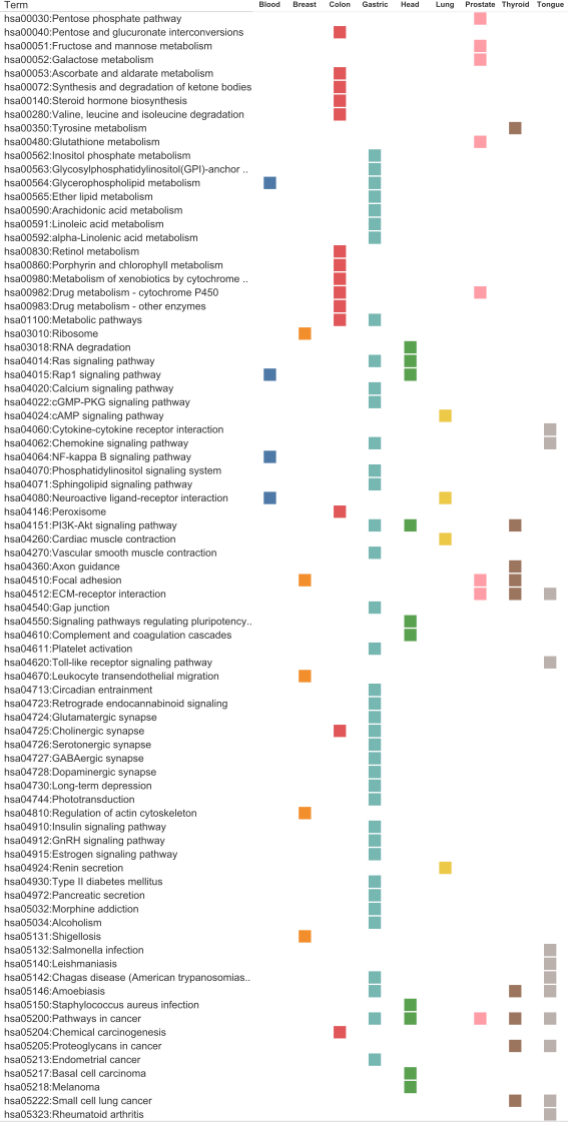
KEGG Pathway mapping for each tissue type using identified genes (potential biomarkers) A pathway was considered significant if its *p*-value was less than or equal to 0.05 and it had a minimum of three tissue-specific genes.

**Figure 8:**
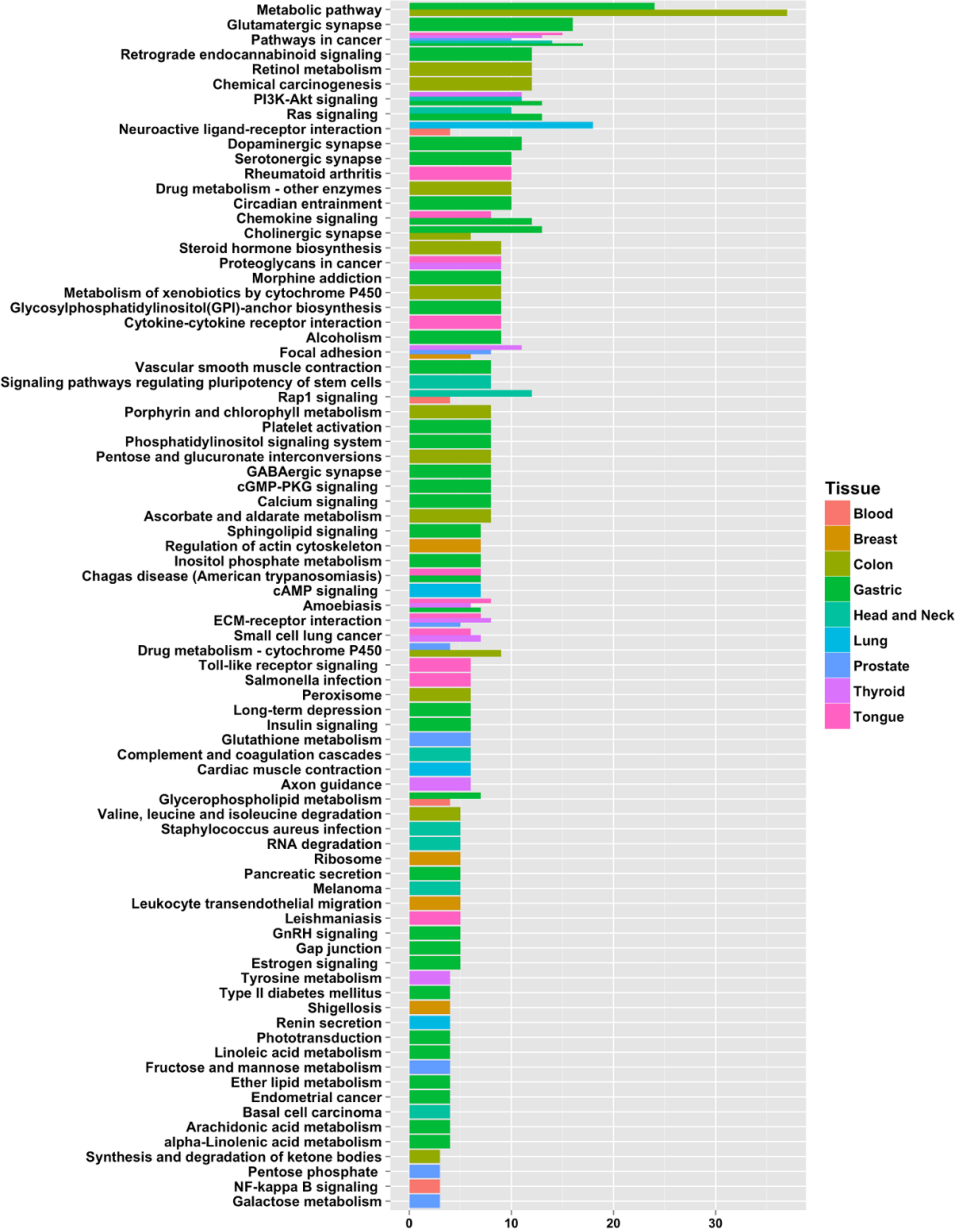
Number of selected genes (potential biomarkers) in pathways for each tissue type A pathway was considered significant if its *p*-value was less than or equal to 0.05 and it had a minimum of three tissue-specific genes.

A total of 104 KEGG (Kyoto Encyclopedia of Genes and Genomes, [35]) pathways were identified for the nine tissue types. The gastric tissue genes had the most pathways (38), whereas the blood and lung tissue genes had the fewest pathways (4). The colon-tissue genes had the second highest number (14) of KEGG pathways (Figures 6-8, Supplementary Figures 4–6, and Supplementary File 4).

Significant pathways for each tissue type are presented below.

#### Blood

Four pathways were identified using blood tissue genes. The only metabolic pathway identified was hsa00564: glycerophospholipid metabolism. The other three pathways are involved in intracellular signaling: hsa04015: Rap1 signaling pathway, hsa04064: NF-kappa B signaling pathway and hsa04080: neuroactive ligand-receptor interaction.

#### Breast

Six pathways were identified using the breast tissue genes. Some of these pathways are involved in inter- or intra-cellular structures: hsa04510: focal adhesion and hsa04810: regulation of the actin cytoskeleton. The rest were signaling motifs: hsa04670: leukocyte transendothelial migration, hsa03010: ribosome and hsa05131: shigellosis pathway.

#### Colon

Colon tissue genes were used to identify 14 pathways. One of this pathways was a signaling pathway: hsa04725: cholinergic synapse. Another pathway was involved in disease development, hsa05204: chemical carcinogenesis. Most of the remaining pathways were involved in diverse metabolic functions: hsa00830: retinol metabolism, hsa00982: drug metabolism–cytochrome P450, hsa00983: drug metabolism–other enzymes, hsa00053: ascorbate and aldarate metabolism, hsa00040: pentose and glucuronate interconversions, hsa00140: steroid hormone biosynthesis, hsa00860: porphyrin and chlorophyll metabolism, hsa00980: metabolism of xenobiotics by cytochrome P450 (For detailed results, refer to the Supplementary File 4).

#### Gastric

Gastric tissue genes were used to identify 38 pathways. Many of the pathways were involved in synaptic function: hsa04724: glutamatergic synapse, hsa04727: GABAergic synapse, hsa04725: cholinergic synapse, hsa04728: dopaminergic synapse, hsa04726: serotonergic synapse. Other pathways were involved in the different signaling aparati (hsa04062: chemokine signaling pathway, hsa04014: ras signaling pathway, hsa04070: phosphatidylinositol signaling system, hsa04151: PI3K-Akt signaling, hsa04071: sphingolipid signaling, hsa04744: phototransduction, and hsa04022: cGMP-PKG signaling pathway). The disease-related pathways included hsa05200: pathways in cancer, hsa05034: alcoholism, hsa05142: Chagas disease, hsa05146: amoebiasis, hsa04930: type II diabetes mellitus, hsa05213: endometrial cancer. The remaining pathways are involved in various forms of fatty acid chain metabolism: hsa00562: inositol phosphate metabolism, hsa00564: glycerophospholipid metabolism, hsa00592: alpha-Linolenic acid metabolism, hsa00565: ether lipid metabolism, hsa00563: glycosylphosphatidylinositol (GPI)-anchor biosynthesis, hsa00590: arachidonic acid metabolism (Supplementary File 4).

#### Head and neck

Using the Head and Neck tissue genes we identified ten pathways. Most of these pathways are specific to cellular signaling and regulation of signaling pathways: hsa04015: Rap1 signaling, hsa04610: complement and coagulation cascades, hsa04550: signaling pathways regulating pluripotency of stem cells, hsa04014: Ras signaling, hsa04151: PI3K-Akt signaling and has03018: RNA degradation. The disease-related pathways involve hsa05150: Staphylococcus aureus infection, hsa05218: melanoma, hsa05200: pathways in cancer, and hsa05217: Basal cell carcinoma.

#### Lung

Four pathways were identified using lung tissue genes. Three of these pathways were involved in signal transduction: hsa04080: neuroactive ligand-receptor interaction, hsa04024: cAMP signaling, and hsa04924: renin secretion. The fourth pathway is involved in hsa04260: cardiac muscle contraction.

#### Prostate

Prostate tissue genes were used to identify eight pathways. These include several metabolic pathways: hsa00480: glutathione metabolism, hsa00051: fructose and mannose metabolism, hsa00982: drug metabolism–cytochrome P450, hsa00030: pentose phosphate pathway and hsa00052: galactose metabolism. The other pathways are hsa04512: ECM-receptor interaction signaling pathway, hsa05200: pathways in cancer, and hsa04510: focal adhesion, a structural pathway.

#### Thyroid

Nine pathways were identified using thyroid tissue genes. The signaling pathways included hsa04512: ECM-receptor interaction and hsa04151: PI3K-Akt signaling. A few structural pathways were identified, including hsa04510: Focal adhesion, hsa05205: proteoglycans in cancer and hsa04360: axon guidance. The only metabolic pathway identified was hsa00350: tyrosine metabolism. The disease-related pathways include hsa05222: small cell lung cancer, hsa05200: pathways in cancer, and hsa05146: amoebiasis.

#### Tongue

Twelve pathways were identified using tongue-tissue genes. Many of the identified pathways were disease-related, including hsa05323: rheumatoid arthritis, hsa05146: amoebiasis, hsa05200: pathways in cancer, hsa05142: Chagas disease, hsa05132: Salmonella infection, hsa05222: small cell lung cancer, and hsa05140: leishmaniasis. The only structural pathway was hsa05205: proteoglycans in cancer. The following four signaling pathways were hsa04620: Toll-like receptor signaling, hsa04062: chemokine signaling, hsa04512: ECM-receptor interaction, and hsa04060: cytokine-cytokine receptor interaction.

#### Enrichment of cancer tissue-specific genes in various functional groups

**Figure 9:**
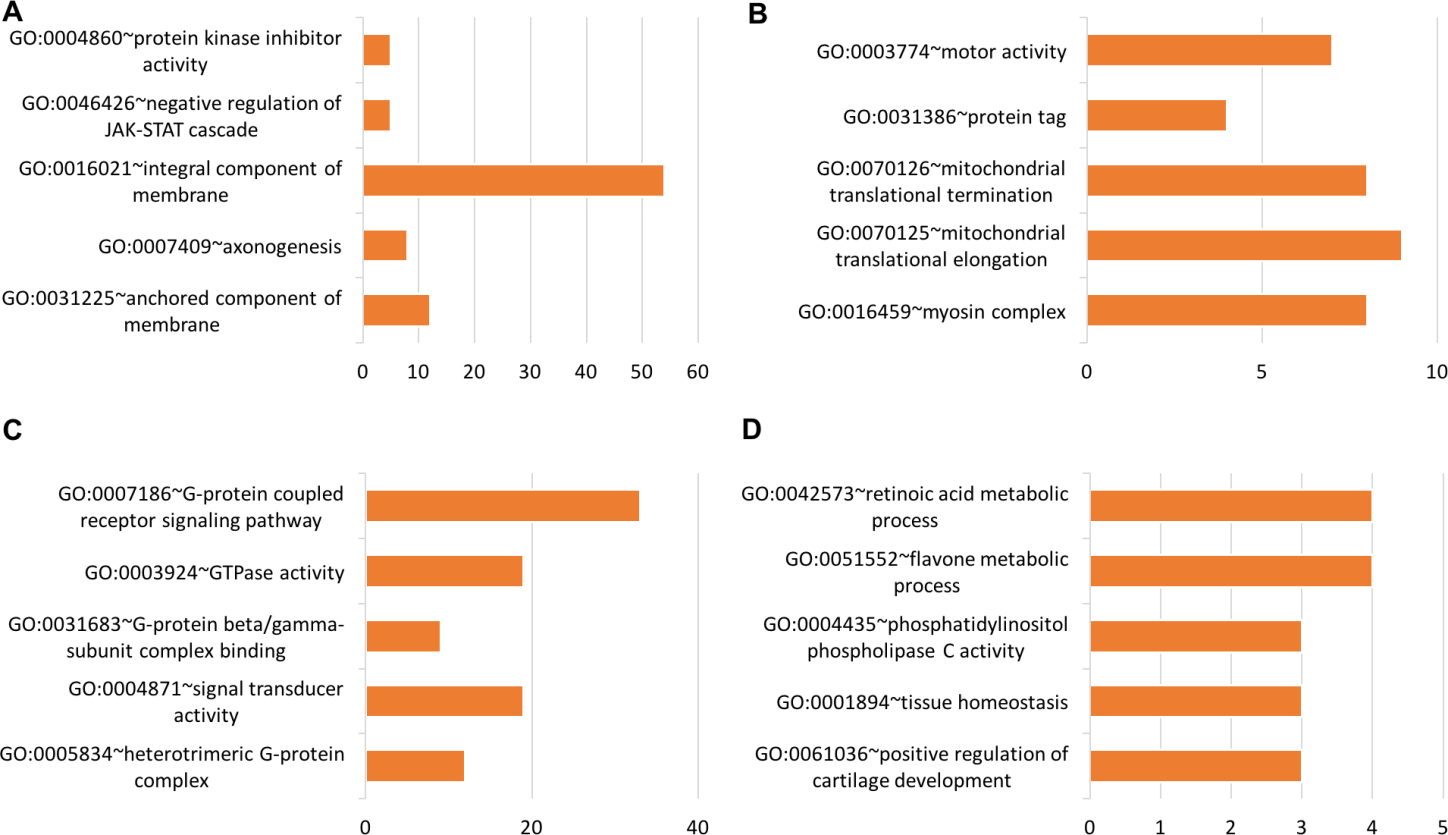
Top five significant Gene Ontology groups with the total number of predicted tissue genes A functional group was considered significant if its *p*-value was less than or equal to 0.05 and if it had a minimum of three tissue-specific genes. (**A**) Blood, (**B**) Breast, (**C**) Colon, (**D**) Gastric

**Figure 10:**
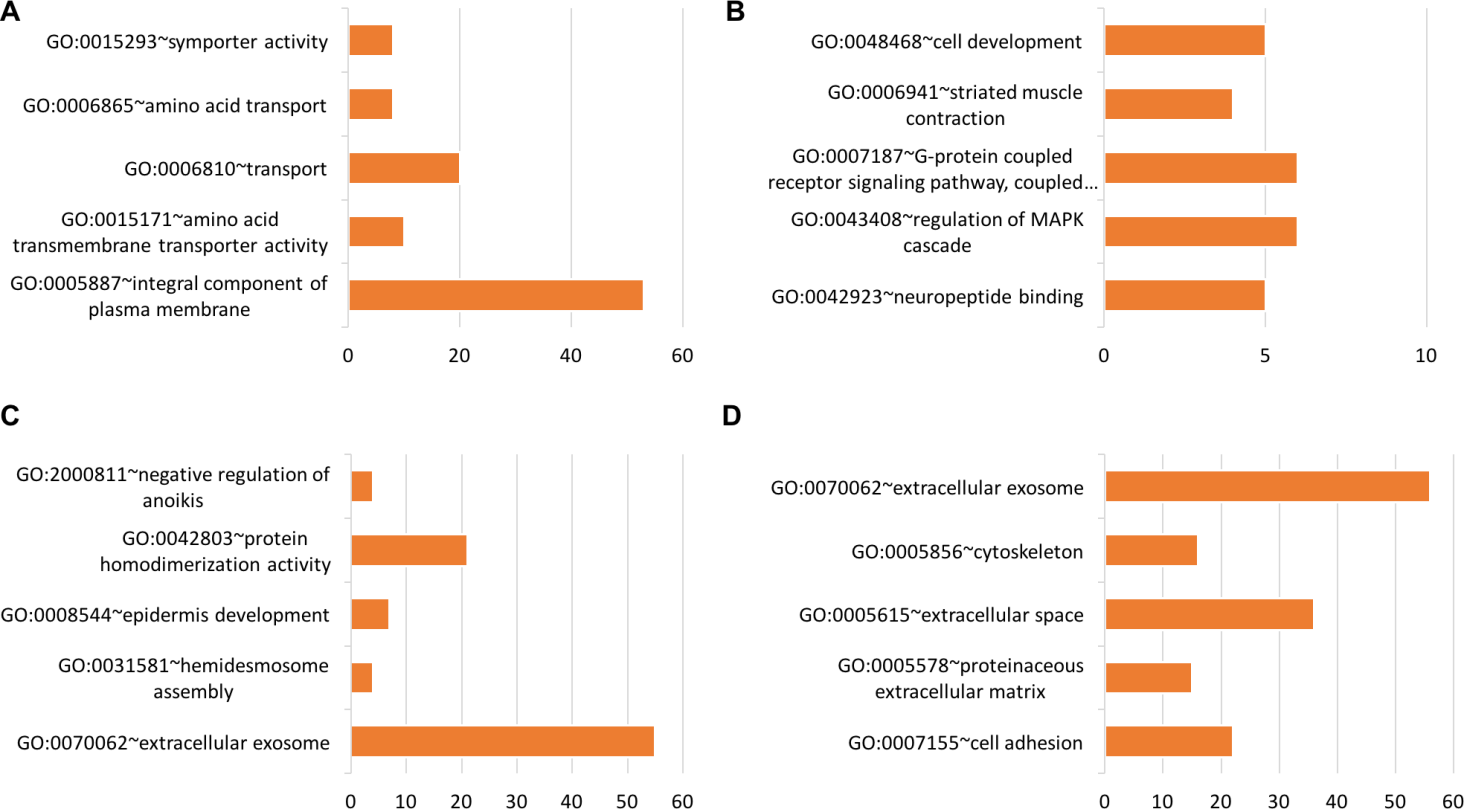
Top five significant Gene Ontology groups with the total number of predicted tissue genes A functional group was considered significant if its *p*-value was less than or equal to 0.05 and it had a minimum of three tissue-specific genes. (**A**) Head & neck, (**B**) Lung, (**C**) Prostate, (**D**) Thyroid

**Figure 11:**
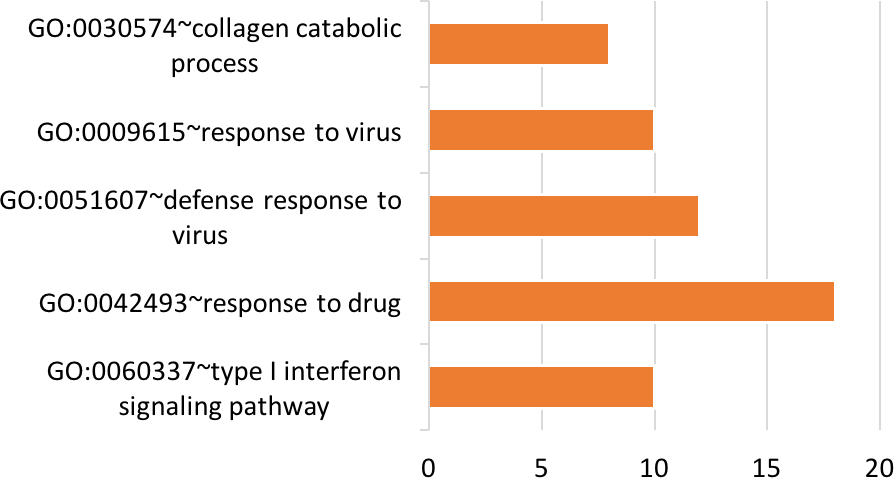
Top five significant Gene Ontology groups with the total number of predicted tongue tissue genes A functional group was considered significant if its *p*-value was less than or equal to 0.05 and if it had a minimum of three tissue-specific genes.

Using tissue-specific genes, functional groups were identified related to protein kinase inhibitor activity (GO:0004860), negative regulation of JAK-STAT cascade (GO:0046426), myosin complex (GO:0016459), G-protein coupled receptor signaling pathway (GO:0007186), GTPase activity (GO:0003924), signal transducer activity (GO:0004871), flavone metabolic process (GO:0051552), tissue homeostasis (GO:0001894), amino acid transmembrane transporter activity (GO:0015171), regulation of MAPK cascade (GO:0043408), type I interferon signaling pathway (GO:0060337), and others. Figures 9–11 show the functional groups with the top five Gene Ontology (GO) groups with the total number of genes from each tissue-specific gene list. See Supplementary File 5 for full list of functional groups.

#### Predicted biomarkers perform better than existing biomarkers

**Figure 12:**
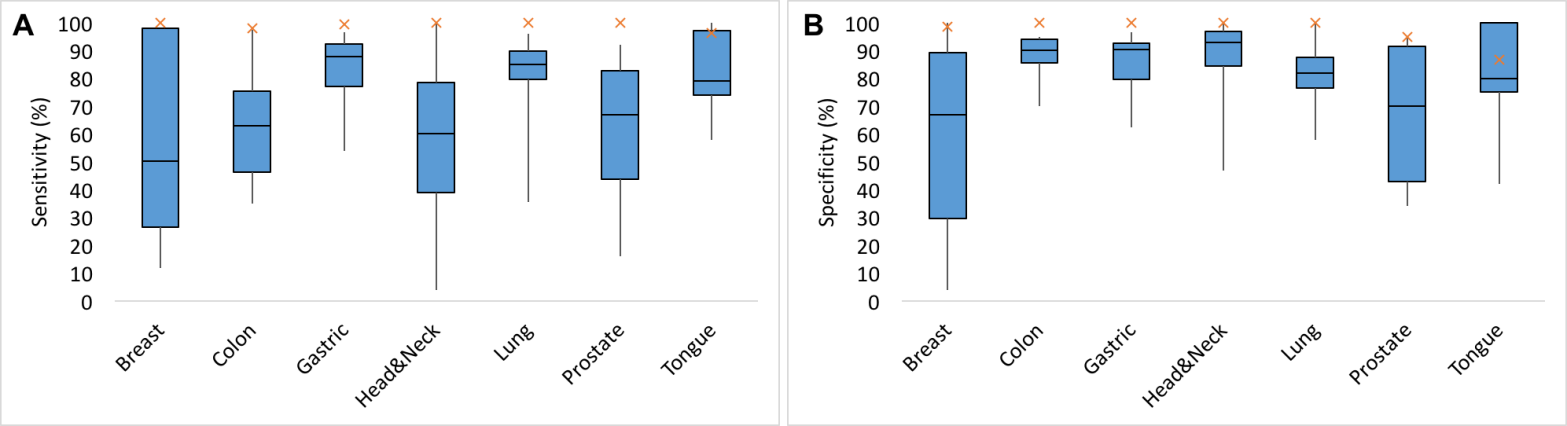
Performance of predicted biomarkers with known biomarkers for each tissue type (**A**) Sensitivity of the existing biomarkers (breast: 50.1%, colon:63%, gastric:87.95%, head&neck:60%, lung:85%; prostate:67%, tongue:79%) is represented by box plot (blue) and sensitivity of our predicted biomarkers (breast:100%, colon:97.92%, gastric: 99.37 %, head & neck:100%, lung:100%; prostate:100%, tongue:96.3%) is represented by cross mark (orange). (**B**) specificity of the existing biomarkers (breast:66.89%, colon:90%, gastric: 90.3%, head& neck:92.9%, lung:82%; prostate:70%, tongue:80%) is represented by box plot (blue) and specificity of our predicted biomarkers (breast:98.46%, colon: 100%, gastric: 100%, head & neck:100%, lung:100%; prostate:95%, tongue:86.67%) is represented by cross (orange).

A total of 244 potential biomarkers were identified for each tissue type distributed across the different cancer types (Supplementary File 2). The quality of these predictions was assessed by comparing the sensitivity and specificity of biomarkers to the sensitivity and specificity of existing biomarkers collected from the literature (Supplementary Tables 10–16). Biomarkers predicted by our machine learning models resulted in higher sensitivity and specificity for each tissue type than those of existing biomarkers (Figure 12).

## DISCUSSION

**Figure 13:**
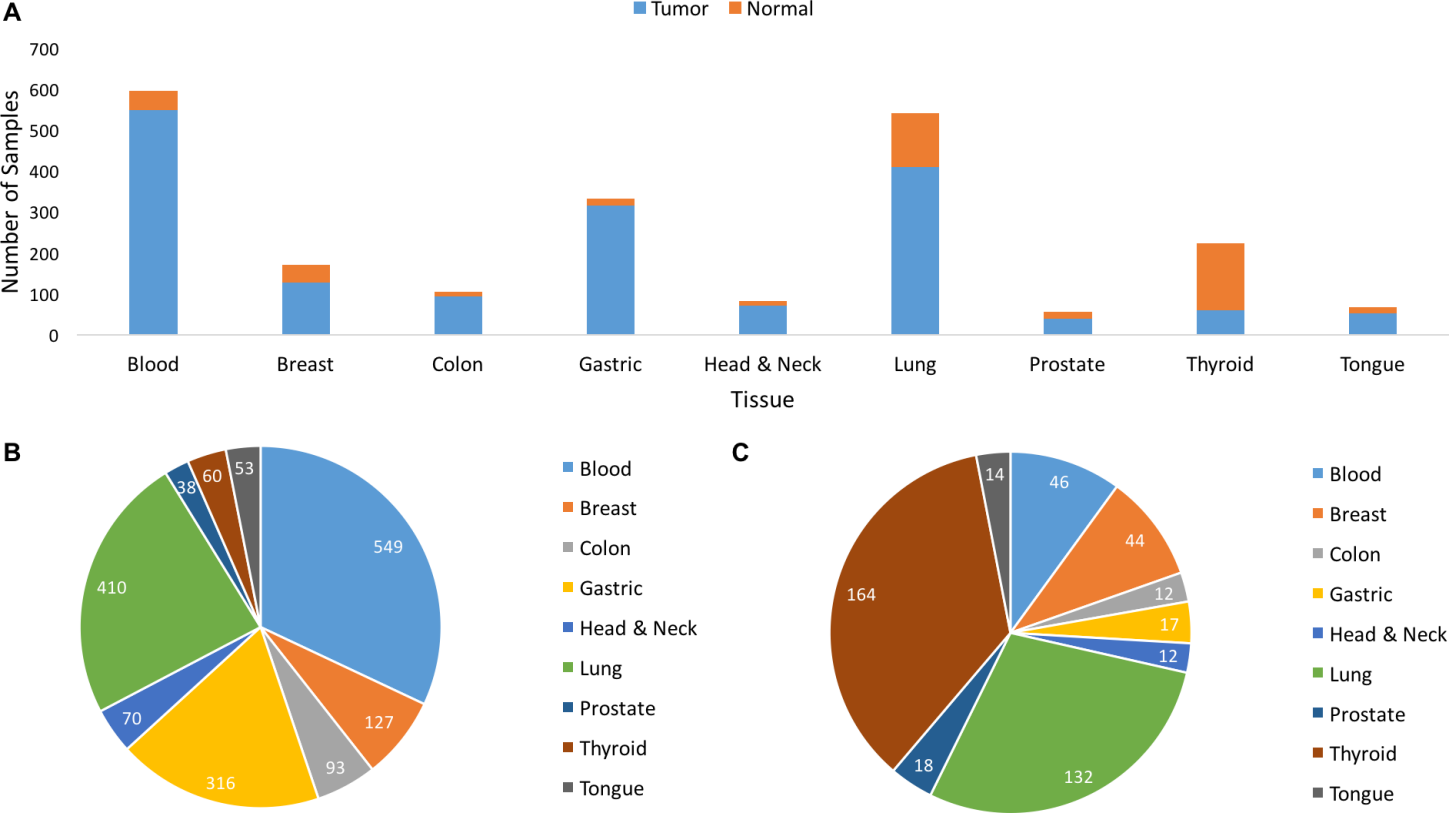
Sample Distribution of Tumor and Normal samples by tissue of origin (**A**) Distribution of Tumor and Normal samples (2175) by tissue of origin. (**B**) Distribution of Tumor (1716) samples by tissue of origin. (**C**) Distribution of Normal (459) samples by tissue of origin.

In this study, machine learning models were developed to analyze a large-scale human gene-expression dataset to identify cancer biomarkers within nine tissue types. Given the presence of cancer, machine learning models were also equipped to distinguish between cancer types. A machine-learning method to select informative genes (potential biomarkers) was identified for each tissue type. Four different classifiers were developed: (1) models which, given a sample of a specific tissue type, accurately classify the sample as cancer or normal (“single-tissue”), (2) a model which, given a sample of any of nine tissue types, accurately classifies the sample as cancer or normal (“multi-tissue bi-class”), (3) a model which, given a sample of any of the nine tissue types, accurately classifies the sample as cancer or normal, and as of a specific tissue type (“multi-tissue multi-class”), and (4) a model which, given a normal sample of any of the nine tissue types, accurately classifies the sample as of a particular tissue type (multi-tissue normal multi-class). (See Figure 13A and Supplementary Table 17 for distribution of samples among tissue types.) The classifiers, trained to incorporate a vast array of different tissue types, and the predicted biomarkers may facilitate accurate, unbiased cancer diagnosis and effective treatment, ultimately improving prognoses.

### Machine learning methodology

The selection of relevant genes involved in different types of cancer remains a challenge [36, 37]. Moreover, for diagnostic purposes, it is important to find a small subset of genes that are sufficiently informative to distinguish between different cancer types. To extract useful gene information from cancer microarray data and reduce dimensionality, feature-selection algorithms were systematically investigated in this study. To this end, a feature selection method was identified (FAER with 1% feature threshold) from twelve feature selection algorithms to select informative genes (potential biomarkers) for each tissue type. As we showed, selecting relatively small subsets of genes significantly improved the performance of our classification models. The single-tissue models were tested using the testing data from all the nine tissues as part of the negative control. Each single-tissue model more accurately classified samples from the same tissue type (same-tissue) than it did samples from other tissue types (across-tissues). The multi-tissue bi-class and multi-class models were not only able to classify the sample as cancer or normal but also the tissue of origin. Moreover, this feature selection process also identified genes that are closely related to the pathways and functional groups of various cancers.

### Metabolic pathways

The metabolism of a tumor depends on both the genotype and tissue of origin and has implications regarding the design of therapies targeting tumor metabolism [38]. Tissue-specific genes pointed to metabolic pathways that may be critical to tumor development in general and tissue-specific tumor development (see the list of pathways in Supplementary File 4). Metabolic rewiring is essential for the progression of many types of cancer [39, 40]. We discuss the metabolic pathways for selected tissues below.

#### Blood

The metabolic pathway identified using the blood tissue genes is glycerophospholipid metabolism, and there is an increase of acyl-glycerophospholipids in acute myeloid leukemia [41].

#### Colon

Most of the metabolic pathways identified using the colon tissue genes have known links to colon cancer. One such pathway is retinol metabolism; retinoids are known to play a role in the prevention and treatment of colorectal cancer [42–44]. Some of the colon-cancer genes identified also include steroid hormone biosynthesis, as the bacterial cells in the gut produce steroid hormones that can have implications for colon cancer [45]. Some colon cancer genes also identified include metabolism of xenobiotics; biotransformation of xenobiotics occurs in the human colon and rectum, and it is known to be associated with colorectal cancer [46–48]. Pentose and glucuronate interconversions were also identified using colon-tissue genes. The heightened metabolic demands of colon cancer cells are known to result in increased glucose uptake and glycolytic flux relative to normal tissues [49, 50]. One common feature of the altered metabolism in cancer is the increased glucose uptake and fermentation of glucose to lactate, a phenomenon known as the Warburg Effect [51, 52]. In tumor cells and other proliferating cells, the rate of glucose uptake dramatically increases, even in the presence of oxygen and fully functioning mitochondria.

#### Gastric

Many of the pathways identified using gastric tissue genes are involved in various forms of fatty acid chain metabolism: inositol phosphate metabolism, glycerophospholipid metabolism, ether lipid metabolism, glycosylphosphatidylinositol (GPI)-anchor biosynthesis, arachidonic acid metabolism and alpha-Linolenic acid metabolism. α-linolenic acid is known be the most effective in suppressing the growth of gastric cancer cells [53]. These results suggest that the metabolism of fatty acids may play a critical role in the tumorigenesis of gastric cancer. Levels of metabolism of fatty acids in cancer cells are known to vary across tissue types [54].

#### Prostate

Some of the metabolic pathways identified using the prostate tissue genes are glutathione metabolism and pentose phosphate metabolism. The glutathione S-transferases (GSTs) enzymes are known to be involved in the metabolism of numerous potential prostate carcinogens [55, 56]. Cancer cells display an increased demand for glucose. Clinical data suggested that the glucose-6-phosphate dehydrogenase (G6PD), the rate-limiting enzyme in the pentose phosphate pathway, is upregulated in prostate cancer [57].

#### Thyroid

Using the thyroid tissue genes, we identified a tyrosine metabolism pathway where the thyroid gland uses tyrosine residues to generate T3 and T4, metabolic hormones known to be involved in thyroid cancer [58, 59].

### Signaling pathways

Signaling pathways controlling cell growth, cell division, cell death, cell fate, and cell motility are almost invariably altered in cancer [60]. Many of the signaling pathways found in this study were identified using tissue-specific genes (discussed below).

#### Blood

The pathways identified using the blood tissue genes are involved in intracellular signaling (Rap1 signaling, NF-kappa B, and neuroactive ligand-receptor interaction signaling). Ras is known to induce myeloproliferative disorders and acute myeloid leukemia [61, 62]. Nuclear factor-kappaB is constitutively activated in human acute myeloid leukemia cells [63–68].

#### Gastric

Signaling pathways and interaction networks were altered in gastric cancer tissues [69, 70]. The pathways identified using the gastric tissue genes were involved in signaling aparati (chemokine signaling, Ras signaling, phosphatidylinositol signaling, PI3K-Akt signaling, sphingolipid signaling, phototransduction, and cGMP-PKG signaling). Phototransductive proteins are expressed to increase intracellular calcium in tumor cells for gastric cancer patients [71, 72].

#### Head and neck

Most of the pathways identified using head and neck genes are specific to cellular signaling and regulation of signaling pathways known to be involved in head and neck cancer, including complement and coagulation cascades, signaling pathways regulating pluripotency of stem cells, Ras signaling, PI3K-Akt signaling and RNA degradation. Rap1 signaling, Rap1, and Rap1GAP are known to play a role in the progression of squamous-cell carcinoma of the head and neck. Rap-1A pathway is also associated with survival, tumor progression, and metastasis of oral cavity squamous cell carcinoma patients [73, 74].

### Infectious disease-related pathways

Many cancers have been attributed to infections [75–77]. Cancers caused by infections are thought to result from one or more of the following: immune suppression, chronic inflammation, and dysregulated inflammation [78–80]. Many of these infectious disease-related pathways were found using the tissue-specific genes identified in this study. For example, the Staphylococcus aureus (gram positive bacteria) pathway was found using head and neck genes. Staphylococcus aureus is known to be present in oral squamous-cell carcinoma tissue [81] and is also abundant in the blood of oral cancer patients [82]. The infectious disease-related pathways identified using the gastric tissue genes include Type II diabetes mellitus and Chagas disease. Type II diabetes mellitus is known to increase the risk of gastric cancer [83]. Chagas disease affects several gastrointestinal regions, but there is no apparent relationship with the growing incidence of cancer [84].

### Gene ontology functional analysis

A Gene Ontology-based similarity assessment indicates that the selected genes for each tissue type are functionally diverse, further validating our gene selection method.

#### Blood

Many of the functional groups identified are known to be involved in cancer. For example, protein-kinase inhibitor activity (GO:0004860) and negative regulation of JAK/STAT cascade (GO:0046426) groups were identified using the blood tissue genes. Tyrosine kinase inhibitors are known to be useful in the treatment of acute myeloid leukemia [85]. The JAK/STAT signaling pathway is a known target for the treatment of leukemia [86].

#### Breast

One of the many functional groups found by the methods of this study was the breast-cancer gene list, which includes the myosin complex (GO:0016459). Myosin is known to promote breast cancer malignancy by enhancing tumor cell proliferation [87]. Mutant p53-associated motor protein myosin upregulation is known to promote breast cancer invasiveness and metastasis [88, 89]. Myosin light-chain kinase is known to play a role in the proliferation and migration of breast cancer cells [90].

#### Colon

A few of the many functional groups found using our colon cancer gene list include the G-protein coupled receptor signaling pathway (GO:0007186) and GTPase activity (GO:0003924). G-protein coupled receptor kinase-5 is known to regulate proliferation and chemokine gene expression in human colon cancer epithelial cells [91]. G-protein-coupled receptors for short-chain fatty acids are known to suppress colon cancer [92]. GTPase activation is known to be present in colon cancer [93].

#### Gastric

The flavone metabolic process (GO:0051552) was identified using gastric tissue genes. Flavone, derived from plants, is known to induce apoptosis in human gastric-cancer cells [94].

#### Lung

Significant functional groups found using lung tissue genes include the G-protein coupled receptor signaling pathway (GO:0007187) and the regulation of MAPK cascade (GO:0043408). The G protein-coupled receptor is known to promote tumorigenesis and is highly expressed in lung cancer [95]. Overexpression of G protein-coupled receptors is known to correlate with poorer tumor differentiation and higher tumor proliferation in non-small-cell lung cancer [96]. Expression of Mitogen-Activated Protein Kinase is known to present in patients with small cell lung cancer [97–100].

### Biomarkers

Biomarkers can be used in clinical settings for patient assessment, estimates of morbidity, screening for cancer, distinguishing benign tissue from malignant tissue, and determination of prognosis. The sensitivity and specificity of biomarkers identified in this study exceeded those of known biomarkers for all compared tissue types, suggesting that these predicted biomarkers are robust indicators of cancer. Further research may include the testing blood-based biomarkers from the list of biomarkers under consideration for this study. For example, blood-based biomarkers have been used for diagnosis, prognosis and treatment of colorectal cancer [7, 101], breast cancer [8, 102], prostate cancer ([103], ovarian cancer [104], and lung cancer [9].

Machine learning cancer prediction models were developed to identify potential biomarkers for unbiased cancer diagnosis and effective treatment, ultimately improving prognoses. Large publicly-available tissue-specific microarray gene expression data were used for cancer type prediction, as well as characterization of tissue-specific normal samples into their various tissues of origin. A logical next step in this work would be the application of machine learning to the generation of a working model of both homeostatic and cancer developmental processes for cancer biomarker detection and early diagnosis. Such work would require collection of numerous forms of data (such as methylation, metabolic and even miRNA data) from a diverse panel of patients including but not limited to, demographic information, normal tissue controls, tumor characteristics, different forms of cancer, subtypes of cancer, and perhaps even other inflammatory diseases such as rheumatoid arthritis, from patients at varying stages of disease progression and development.

## MATERIALS AND METHODS

### Data collection

Microarray gene expression data were collected from NCBI Gene Expression Omnibus (GEO) repository [105]. A total of 2,175 tissue samples, both normal and cancerous, were collected from nine distinct tissues: blood (595), breast (171), colon (105), gastric (333), head and neck (82), lung (542), prostate (56), thyroid (224), and tongue (67). The detailed sample distribution is shown in Figure 13, and Supplementary Table 16. The accession numbers for the data are as follows: blood data: GSE6891, GSE267, GSE43346, GSE63270; breast data: GSE5460, GSE2361, GSE20437, GSE43346; colon data: GSE64857, GSE4107, GSE2361, GSE43346; gastric data, GSE2361, GSE43346, GSE19826, GSE62254, GSE8167; head and neck data: GSE45153, GSE10300, GSE43346, GSE8987; lung data: GSE1133, GSE10072, GSE2361, GSE43346, GSE16538, GSE19804, GSE21369, GSE24206, GSE63074; prostate data: GSE46602, GSE6369, GSE1133, GSE2361, GSE43346; thyroid data: GSE33630, GSE5054, GSE58545, GSE2361, GSE43346, GSE60542, GSE3467, GSE3678, GSE35570; tongue data: GSE52915, GSE9844, GSE1133, GSE43346. Samples used in this study were collected directly from patients according to experimental design. The frequency of data derived from tissue samples was balanced across tissue classes and entered into a composite data set. The data were collected from the following three Affymetrix Human Genome: HG-U133_Plus_2, HG-U133A, and HG-U133A_2.

### Normalization and background correction

Normalization and preprocessing are essential steps for the analyses of high-throughput data including microarrays. The Affy R module 1.54 [106] from Bioconductor package (https://bioconductor.org/packages/release/bioc/html/affy.html) was used to remove the technical variation from noisy data and background noise from signal intensities. The Quantile Normalization Method [107] was used to normalize the data, and the background correction was performed using the Robust Multi-Average (RMA) [108] parameter method. Quantile normalization method relies on the assumption that observed global changes across samples are due to unwanted technical variability. We used quantile normalization since it is a simple, fast, one-size-fits-all solution for transforming all the arrays to have a common distribution of intensities. The algorithm maps every value on any one chip to the corresponding quantile of the standard distribution. The intensities of all probes on each chip into one standard distribution shape, which is determined by pooling all the individual chip distributions. We used RMA because it has a smaller standard deviation at all levels of expression compared to dChip and MAS5.0 [108].

### Probe to gene mapping

Using the information provided in Affymetrix annotation files (http://www.affymetrix.com/support/technical/annotationfilesmain.affx), probe names were replaced with their respective gene names. Since multiple probes can also correspond to the same gene, the expression values for duplicate entries were averaged within samples. All preprocessed data were randomly divided into equal-sized subsets of training and testing datasets. Since the datasets are unbalanced across classes, class distributions are approximately preserved for each tissue using stratified partitioning for training and testing sets.

### Identification of best feature selection algorithm

The key to construction of accurate and unbiased machine learning models from microarray gene expression data is identification of the features (genes) best able to predict tissue class and cancer status [109]. The test set must be kept separate from the model training set Support Vector Machine (SVM) [110], IBk K-nearest neighbor [111], and Naive Bayes [112] were used to identify the best feature selection algorithm. The following 12 feature selection algorithms were used to create the models: (Chi Squared_Ranker, ClassifierSubsetEvaluator_GeneticSearch,ConsistencySubsetEvaluator_BestFirst, ConsistencySubsetEvaluator_GeneticSearch,ConsistencySubsetEvaluator_LinearFWDSelection, FilteredAttributeEvaluator_Ranker, GainRatioAttributeEvaluator_Ranker, LatentSemanticAnalysis_Ranker, OneRAttributeEvaluator_Ranker, ReliefFAttributeEvaluator_Ranker, SymmetricalUncertAttributeEval_Ranker, WrapperSubsetEval_GeneticSearch) and 13 feature thresholds (Top 1%, 2%, 3%, 4%, 5%, 10%, 20%, 25%, 33%, 50%, 66%, 75%, 100%) is shown.

### Machine learning classification model construction

**Figure 14:**
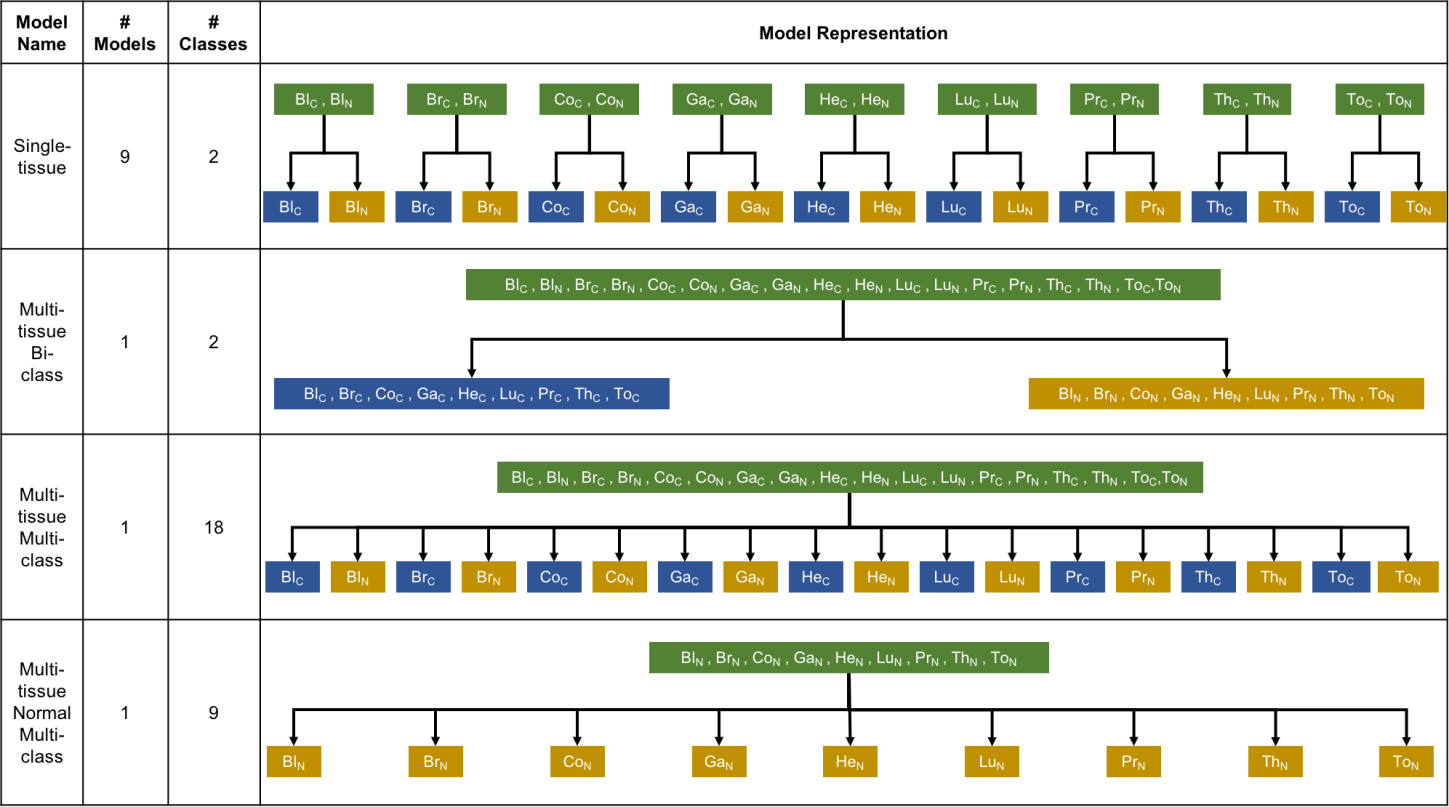
Types of machine learning classification model construction with the model name, the total number of models, the number of classes, and disease states of sample source for each model type Green box: unlabeled data; blue box: cancer label; yellow box: normal label; Bl_C_: blood-cancer, Bl_N_: blood-normal, Br_C_: breast-cancer, Br_N_: breast-normal, Co_C_: colon-cancer, Co_N_: colon-normal, Ga_C_: gastric-cancer, Ga_N_: gastric-normal, He_C_: head and neck-cancer, He_N_: head and neck-normal, Lu_C_: lung-cancer, Lu_N_: lung-normal, Pr_C_: prostate-cancer, Pr_N_: prostate-normal, Th_C_: thyroid-cancer, Th_N_: thyroid-normal, To_C_: tongue-cancer, To_N_: tongue-normal.

Machine learning classification models can be categorized into the following four groups: (1) models which, given a sample of a specific tissue type, classify the sample as cancer or normal (“single-tissue”), (2) models which, given a sample of any of the nine tissue types, classify the sample as cancer or normal (“multi-tissue bi-class”) (3) models which, given a sample of any of the nine tissue types, classifies the sample as cancer or normal and as of a specific tissue type (“multi-tissue multi-class”) and (4) a model which, given a normal sample of any of the nine tissue types, classifies the sample as of a particular tissue type (“multi-tissue normal multi-class”). (See Figure 13A and Supplementary Table 17 for distribution of samples among tissue types). The overall workflow of the model construction is given in Figure 14. Models were constructed using Random Forests and Support Vector Machine. The configurable CancerDiscover software pipeline [113] was used to perform all the machine learning steps in this study.

### Machine learning algorithms and framework

Support Vector Machines (SVMs) and Random Forests were used to construct the models for this study. These machine-learning methods were chosen because of their extensive and successful applications to datasets from genomic and proteomic domains [114, 115]. Some of the cancer classification tasks were binary (two classes), and the others were multiclass (more than two classes). Though SVMs are designed for binary classification, they can also be used for multiclass classification by a one-versus-rest approach [116]. The one-versus-rest approach for classification is known to be among the best-performing methods for multicategory classification for microarray gene expression [30]. Models were also constructed using Random Forests (RF), which can solve multicategory problems natively through direct application.

The Random Forests algorithm is well suited to the classification of genomic data because of the following advantages (i) it performs embedded feature selection (ii) it incorporates interactions between predictors: (iii) it allows the algorithm to accurately learn both simple and complex classification functions; (iv) it is applicable to both binary and multicategory classification tasks [117]. Feature selection and model construction was also accomplished using WEKA (Waikato Environment for Knowledge Analysis) [118] version 3.8.

### Measures

Accuracy was defined as the overall ability of models to categorize testing sample data correctly. Reported measures included the numbers of *true positives* (TP), *true negatives* (TN), *false positives* (FP), and *false negatives* (FN). A true-positive count is the number of samples in a dataset which were correctly categorized *into* classes. A false-positive count is the number of samples in a dataset which were sorted into the wrong category. A true negative count represents the number of samples which were *not* classified into a class to which they do *not* belong, and false negatives are samples which are *not* classified into the class to which they do belong.

Accuracy, Sensitivity (or Recall), Specificity, Precision, and F1-score are derived from the measures mentioned above as follows: accuracy is the ratio of correctly predicted samples to the total number of samples. Sensitivity is the proportion of true positives that are predicted as positives. Specificity is the proportion of true negatives which are predicted as negatives, and Precision is the ratio of true positives to the total number of true negatives and true positives. Lastly, F1-score is defined as the harmonic mean of Precision and Recall and is calculated by first multiplying precision and recall values, then dividing the resulting value by the total of precision and recall, and finally, multiplying the result by two. The Accuracy, Sensitivity, Specificity, Precision, and F1-Score are given by:

$$Accuracy=\frac{TP+TN}{TP+TN+FP+FN}$$

$$\mathrm{Recall}/Sensitivity=\frac{TP}{TP+FN}$$

$$Specificity=\frac{TN}{TN+FP}$$

$$\mathrm{Precision=}\frac{TP}{TP+FP}$$

$$F1-Score=2*\frac{\mathrm{Precision*Recall}}{\mathrm{Precision+Recall}}$$

### Model selection and accuracy estimation

For model selection and accuracy estimation, we used 10-fold cross-validation [30, 115]. This technique separates data into ten parts and uses nine parts for the model generation while predictions are generated and evaluated by using the one part. This step is subsequently repeated ten times, so each part (internal test set) is tested against the other nine parts (internal train set). The average performance over the ten accuracies is accepted as an unbiased estimate of the model’s performance.

### Functional analysis

We used Database for Annotation, Visualization, and Integrated Discovery (DAVID) v6.8 [119] for functional analysis. For each of the nine tissue type provided to DAVID, the tissue-specific gene list consisting of top 244 (1%) of genes were used to classify samples of a particular tissue type as either cancerous or normal (See Supplementary File 2). Within DAVID, KEGG was chosen for pathway analysis. Of the pathways returned, only those with a *p*-value of less than or equal to 0.05 and with three or more of our genes were considered. Within DAVID, Functional Annotation analysis was used for sorting the genes according to functional groups. Of the functional groups returned, only those with a *p*-value of less than or equal to 0.05 and with three or more of the genes identified in this study were considered.

## SUPPLEMENTARY MATERIALS FIGURES AND TABLES















## Abbreviations

FAER: Filtered Attribute Evaluator, Ranker
WEKA: Waikato Environment for Knowledge Analysis
DAVID: Database for Annotation, Visualization, and Integrated Discovery
KEGG: Kyoto Encyclopedia of Genes and Genomes
ML: Machine Learning
SVM: Support Vector Machine
RF: Random Forests
GEO: Gene Expression Omnibus
RMA: Robust Multi Average
GO: Gene Ontology
TP: True Positives
TN: True Negatives
FP: False Positives
FN: False Negatives
ROC: Receiver Operating Characteristics
