# Supplementary material for: Identification of potential tissue-specific cancer biomarkers and development of cancer versus normal genomic classifiers
Source: Oncotarget. 2017 Sep 21;8(49):85692–715. doi: 10.18632/oncotarget.21127 (PMC5689641; doi:10.18632/oncotarget.21127)
Supplement: Supplementary file 1 [file oncotarget-08-85692-s001.pdf]

# Identification of potential tissue-specific cancer biomarkers and development of cancer versus normal genomic classifiers

## SUPPLEMENTARY MATERIALS

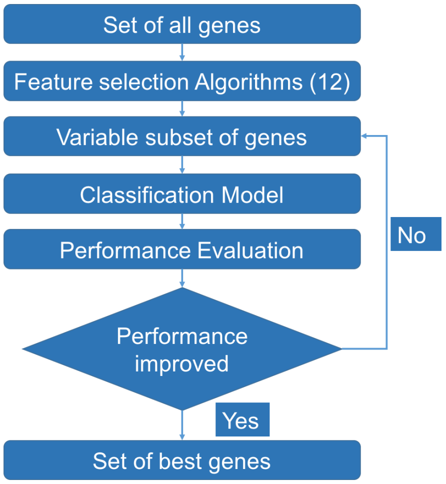


**Supplementary Figure 1: Workflow for identification of the Best Feature Selection Algorithm.** Set of all genes from a specific tissue is given to Feature Selection Algorithms that select the variable subset of genes based on expression values and then build classification model to differentiate a normal sample from a cancer sample. The performance of the model is then evaluated and the process is repeated until the set of best genes are identified.

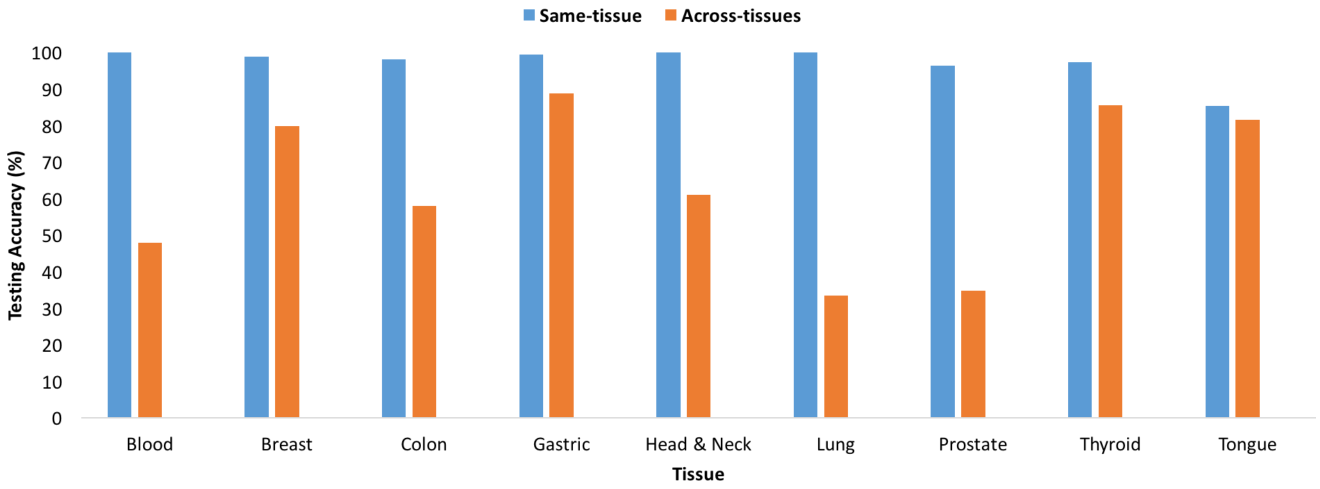


**Supplementary Figure 2: Testing accuracies for single-tissue and across-tissue models.** The testing accuracy of each model for testing data comprised of samples from the same tissue (blue bars) and testing accuracy of each model for testing data composed of samples from all tissues (orange bars)

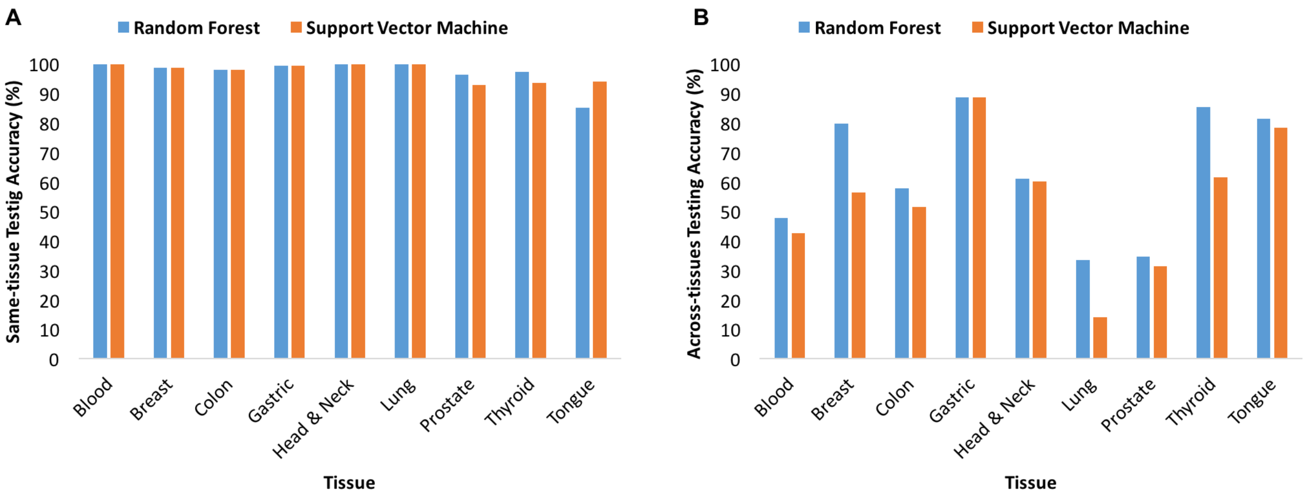


**Supplementary Figure 3: Performance of Random Forests and Support Vector Machine for each single-tissue model.** (**A**) Testing accuracy of each model using testing data comprised of samples from the same tissue for the Random Forests classifier (Blue bars) and the Support Vector Machine classifier (Orange bars). (**B**) Testing accuracy of each model using testing data comprised of samples from all tissues for the Random Forests classifier (Blue bars) and the Support Vector Machine classifier (Orange bars).

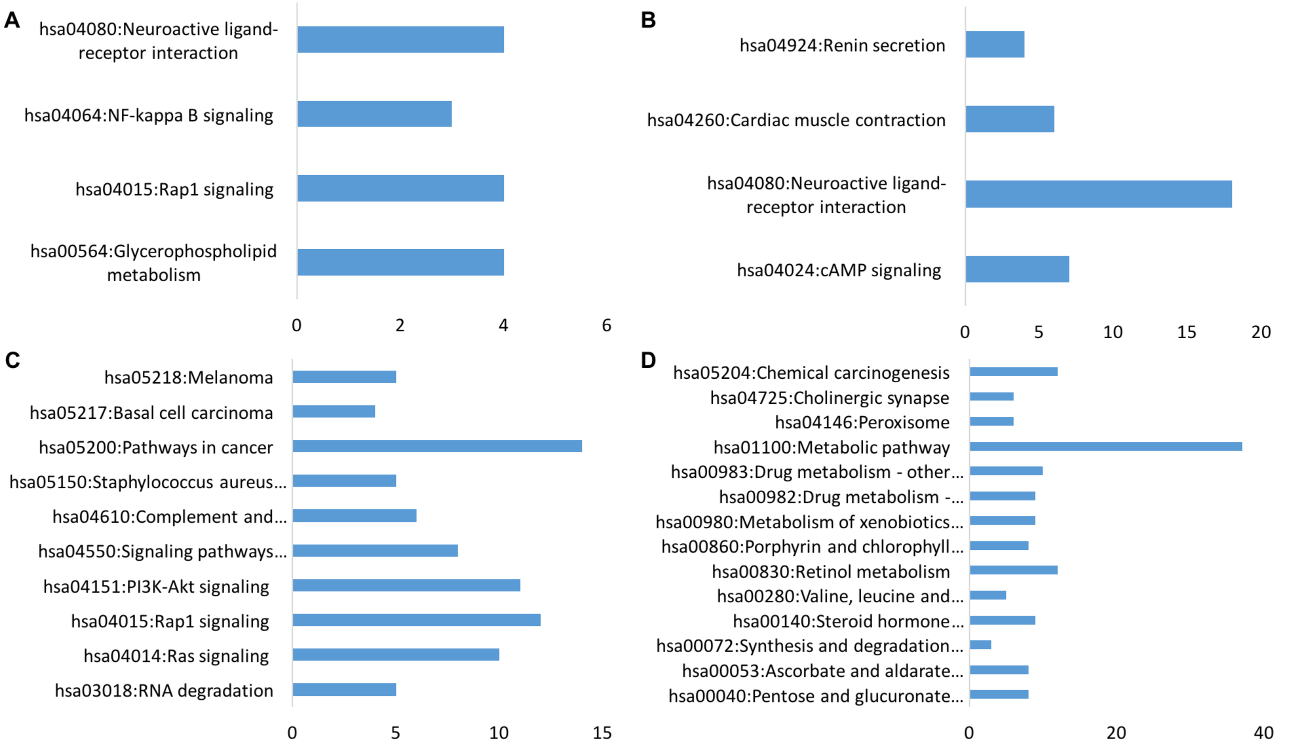


**Supplementary Figure 4: Number of selected features (potential biomarkers) in pathways for each tissue type.** (**A**) Blood (**B**) Lung (**C**) Head and Neck (**D**) Colon. We considered a pathway significant if its p-value was less than or equal to 0.05 and it had a minimum of 3 of our tissue-specific features.

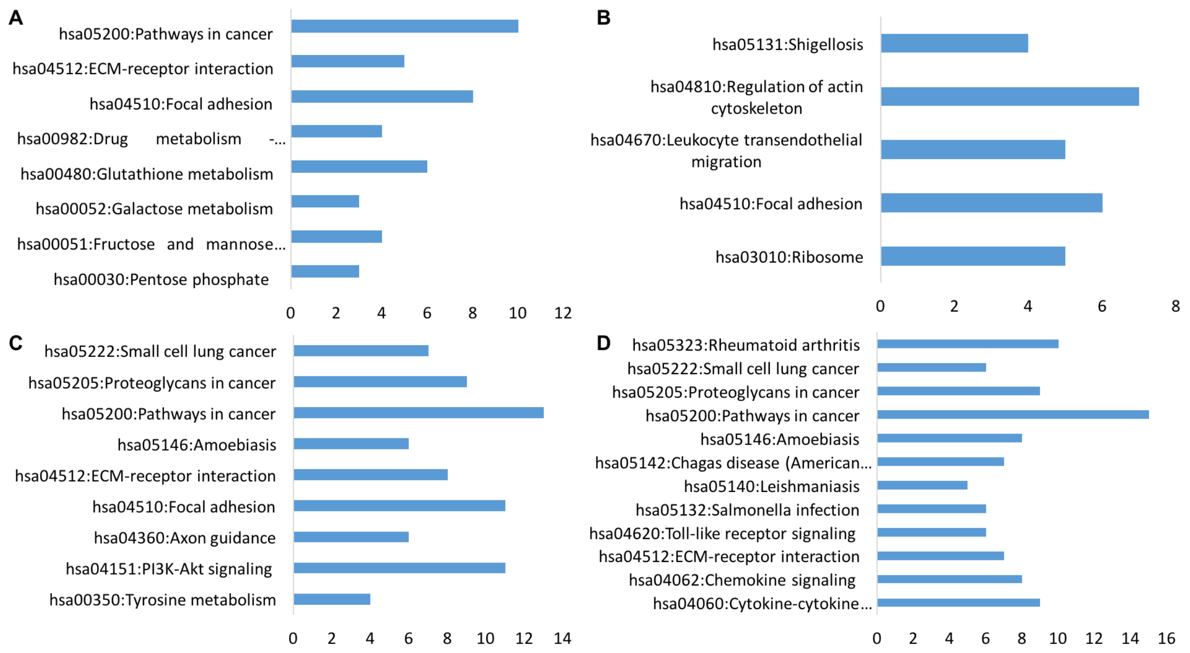


**Supplementary Figure 5: Number of selected features (potential biomarkers) in pathways for each tissue type.** (**A**) Prostate (**B**) Breast (**C**) Thyroid (**D**) Tongue. We considered a pathway significant if its *p*-value was less than or equal to 0.05 and it had a minimum of 3 of our tissue-specific features.

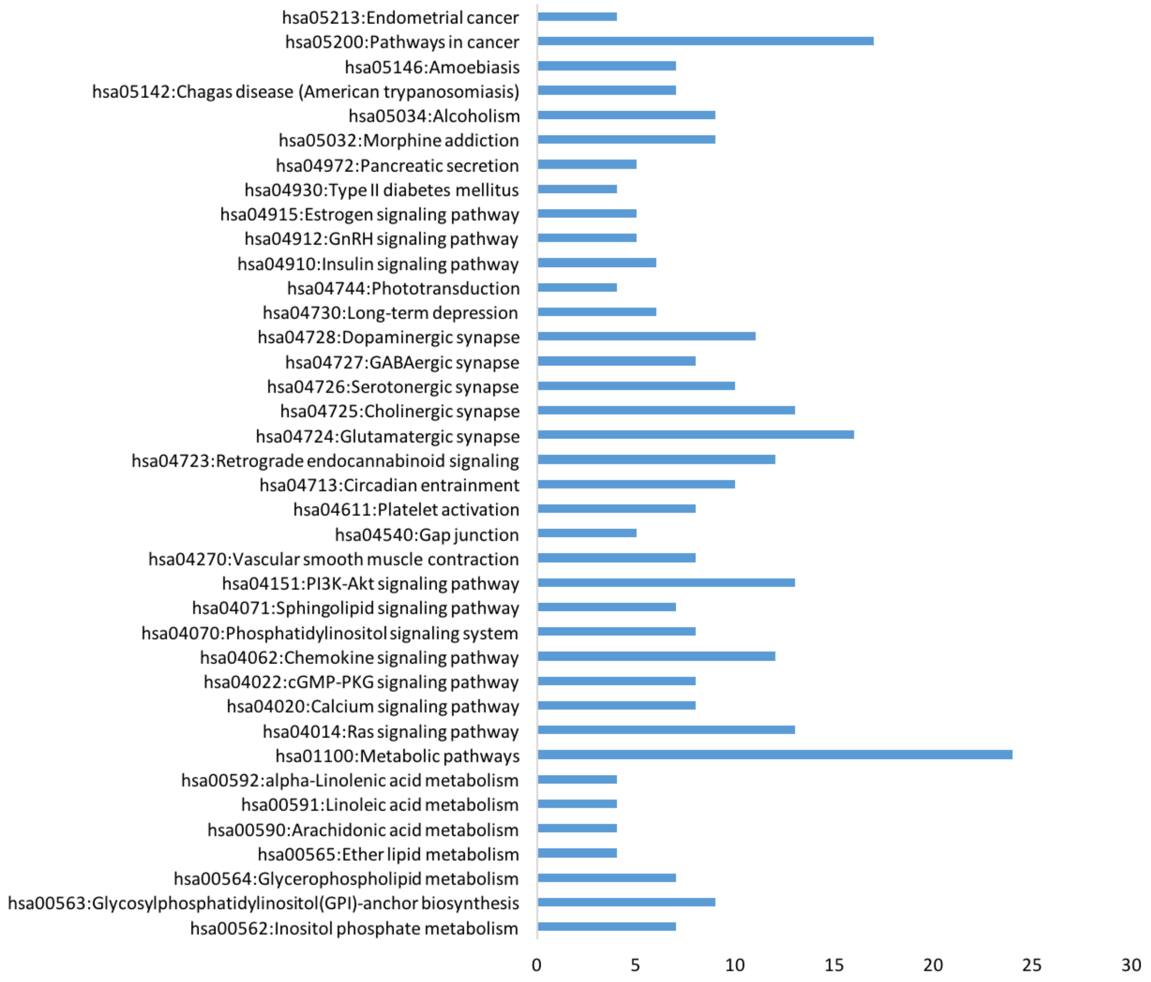


**Supplementary Figure 6: Number of selected features (potential biomarkers) in pathways for Gastric tissue type.** We considered a pathway significant if its p-value was less than or equal to 0.05 and it had a minimum of 3 of our tissue-specific features.

**Supplementary File 1: The performance of various feature selection algorithms and thresholds were tested with SVM, Naïve Bayes and IBk classifiers. The following 12 feature selection algorithms (Chi Squared_Ranker, ClassifierSubsetEvaluator_GeneticSearch, ConsistencySubsetEvaluator_BestFirst, ConsistencySubsetEvaluator_GeneticSearch,ConsistencySubsetEvaluator_LinearFWDSelection, FilteredAttributeEvaluator_Ranker, GainRatioAttributeEvaluator_Ranker, LatentSemanticAnalysis_Ranker, OneRAttributeEvaluator_Ranker, ReliefFAttributeEvaluator_Ranker, SymmetricalUncertAttributeEval_Ranker, WrapperSubsetEval_GeneticSearch) and 13 feature thresholds (1%, 2%, 3%, 4%, 5%, 10%, 20%, 25%, 33%, 50%, 66%, 75%, 100%) is shown. Filtered Attribute Evaluator (ranker method, FAER) using 1% feature threshold is the best performing feature selection algorithm.** See Supplementary_File_1

**Supplementary File 2: The list of predicted biomarkers for each of the nine tissue types.** See Supplementary_File_2

**Supplementary File 3: The list of common predicted biomarkers (genes present in at least 2 tissue type).** See Supplementary_File_3

**Supplementary File 4: The list of KEGG pathways for each tissue type.** See Supplementary_File_4

**Supplementary File 5: The list of Gene Ontology functional groups for each tissue type.** See Supplementary_File_5

**Supplementary Table 1: Testing accuracies for single-tissue models**

| Tissue | Same-tissue testing accuracy | Across-tissues testing accuracy |
|---|---|---|
| Blood | 100 | 47.85 |
| Breast | 98.84 | 79.78 |
| Colon | 98.11 | 57.96 |
| Gastric | 99.4 | 88.68 |
| Head and Neck | 100 | 61.01 |
| Lung | 100 | 33.46 |
| Prostate | 96.43 | 34.75 |
| Thyroid | 97.32 | 85.49 |
| Tongue | 85.29 | 81.44 |

Testing accuracy of each model for testing data comprised of samples from the same tissue and Testing accuracy of each model for testing data composed of samples from all tissues.

**Supplementary Table 2: Performance of random forest and support vector machine for each single-tissue model**

| Tissue | Random Forest | Support Vector Machine |
|---|---|---|
| Blood | 100 | 100 |
| Breast | 98.83 | 98.83 |
| Colon | 98.11 | 98.11 |
| Gastric | 99.40 | 99.40 |
| Head and Neck | 100 | 100 |
| Lung | 100 | 100 |
| Prostate | 96.42 | 92.85 |
| Thyroid | 97.32 | 93.75 |
| Tongue | 85.29 | 94.11 |

Testing accuracy of each model using testing data comprised of samples from the same tissue for the Random Forest classifier and the Support Vector Machine classifier. Testing accuracy of each model using testing data composed of samples from all tissues for the Random Forest classifier and the Support Vector Machine classifier

**Supplementary Table 3: Performance of random forest and support vector machine for each single-tissue model**

| Tissue | Random Forest | Support Vector Machine |
|---|---|---|
| Blood | 47.85 | 42.55 |
| Breast | 79.78 | 56.47 |
| Colon | 57.95 | 51.49 |
| Gastric | 88.67 | 88.67 |
| Head and Neck | 61.01 | 60.15 |
| Lung | 33.46 | 14.14 |
| Prostate | 34.74 | 31.35 |
| Thyroid | 85.48 | 61.68 |
| Tongue | 81.44 | 78.45 |

Testing accuracy of each model using testing data comprised of samples from all tissues for the Random Forest classifier and the Support Vector Machine classifier.

**Supplementary Table 4: Performance of the multi-tissue multi-class model for each class**

| | Training | | | Testing | | |
|---|---|---|---|---|---|---|
| **Class** | **Precision** | **Recall** | **F1-Score** | **Precision** | **Recall** | **F1-Score** |
| Blood-tumor | 100 | 99.63 | 99.82 | 100 | 100 | 100 |
| Blood-normal | 100 | 100 | 100 | 100 | 92 | 95.83 |
| Breast-tumor | 100 | 95.52 | 97.71 | 100 | 100 | 100 |
| Breast-normal | 95.45 | 100 | 97.67 | 100 | 100 | 100 |
| Colon-tumor | 95.65 | 93.61 | 94.62 | 95.74 | 93.75 | 94.74 |
| Colon-normal | 33.33 | 50 | 40 | 33.33 | 50 | 40 |
| Gastric-tumor | 100 | 99.37 | 99.68 | 100 | 100 | 100 |
| Gastric-normal | 100 | 100 | 100 | 88.88 | 100 | 94.12 |
| Head and Neck-tumor | 100 | 100 | 100 | 100 | 100 | 100 |
| Head and Neck-normal | 83.33 | 100 | 90.91 | 66.66 | 100 | 80 |
| Lung-tumor | 100 | 100 | 100 | 100 | 100 | 100 |
| Lung-normal | 100 | 100 | 100 | 100 | 97.05 | 98.51 |
| Prostate-tumor | 84.21 | 76.19 | 80 | 89.47 | 85 | 87.18 |
| Prostate-normal | 33.33 | 14.28 | 20 | 55.55 | 25 | 34.48 |
| Thyroid-tumor | 83.33 | 83.33 | 83.33 | 96.66 | 85.29 | 90.66 |
| Thyroid-normal | 93.9 | 93.9 | 93.9 | 96.34 | 98.75 | 97.53 |
| Tongue-tumor | 96.3 | 81.25 | 88.14 | 92.3 | 77.41 | 84.21 |
| Tongue-normal | 28.57 | 100 | 44.44 | 0 | 0 | 0 |

Precision, Recall and F1-Score using the training and the testing datasets.

**Supplementary Table 5: Confusion matrix for multi-tissue multi-class model (Training set)**

| a | b | c | d | e | f | g | h | i | j | k | l | m | n | o | p | q | r | Classified as |
|---|---|---|---|---|---|---|---|---|---|---|---|---|---|---|---|---|---|---|
| 274 | 0 | 0 | 0 | 0 | 0 | 0 | 0 | 0 | 0 | 0 | 0 | 0 | 0 | 0 | 0 | 0 | 0 | a = Blood-tumor |
| 0 | 23 | 0 | 0 | 0 | 0 | 0 | 0 | 0 | 0 | 0 | 0 | 0 | 0 | 0 | 0 | 0 | 0 | b = Blood-normal |
| 0 | 0 | 64 | 0 | 0 | 0 | 0 | 0 | 0 | 0 | 0 | 0 | 0 | 0 | 0 | 0 | 0 | 0 | c = Breast-tumor |
| 0 | 0 | 0 | 21 | 0 | 0 | 1 | 0 | 0 | 0 | 0 | 0 | 0 | 0 | 0 | 0 | 0 | 0 | d = Breast-normal |
| 0 | 0 | 0 | 0 | 44 | 2 | 0 | 0 | 0 | 0 | 0 | 0 | 0 | 0 | 0 | 0 | 0 | 0 | e = Colon-tumor |
| 0 | 0 | 0 | 0 | 3 | 2 | 0 | 0 | 0 | 0 | 0 | 0 | 0 | 0 | 0 | 0 | 1 | 0 | f = Colon-normal |
| 0 | 0 | 0 | 0 | 0 | 0 | 158 | 0 | 0 | 0 | 0 | 0 | 0 | 0 | 0 | 0 | 0 | 0 | g = Gastric-tumor |
| 0 | 0 | 0 | 0 | 0 | 0 | 0 | 8 | 0 | 0 | 0 | 0 | 0 | 0 | 0 | 0 | 0 | 0 | h = Gastric-normal |
| 0 | 0 | 0 | 0 | 0 | 0 | 0 | 0 | 35 | 0 | 0 | 0 | 0 | 0 | 0 | 0 | 0 | 0 | i = HnN-tumor |
| 0 | 0 | 1 | 0 | 0 | 0 | 0 | 0 | 0 | 5 | 0 | 0 | 0 | 0 | 0 | 0 | 0 | 0 | j = HnN-normal |
| 0 | 0 | 0 | 0 | 0 | 0 | 0 | 0 | 0 | 0 | 205 | 0 | 0 | 0 | 0 | 0 | 0 | 0 | k = Lung-tumor |
| 0 | 0 | 0 | 0 | 0 | 0 | 0 | 0 | 0 | 0 | 0 | 66 | 0 | 0 | 0 | 0 | 0 | 0 | l = Lung-normal |
| 0 | 0 | 1 | 0 | 0 | 0 | 0 | 0 | 0 | 0 | 0 | 0 | 16 | 2 | 0 | 0 | 0 | 0 | m = Prostate-tumor |
| 1 | 0 | 0 | 0 | 0 | 0 | 0 | 0 | 0 | 0 | 0 | 0 | 5 | 3 | 0 | 0 | 0 | 0 | n = Prostate-normal |
| 0 | 0 | 0 | 0 | 0 | 0 | 0 | 0 | 0 | 0 | 0 | 0 | 0 | 0 | 25 | 5 | 0 | 0 | o = Thyroid-tumor |
| 0 | 0 | 0 | 0 | 0 | 0 | 0 | 0 | 0 | 0 | 0 | 0 | 0 | 0 | 5 | 77 | 0 | 0 | p = Thyroid-normal |
| 0 | 0 | 1 | 0 | 0 | 0 | 0 | 0 | 0 | 0 | 0 | 0 | 0 | 0 | 0 | 0 | 26 | 0 | q = Tongue-tumor |
| 0 | 0 | 0 | 0 | 0 | 0 | 0 | 0 | 0 | 0 | 0 | 0 | 0 | 0 | 0 | 0 | 5 | 2 | r = Tongue-normal |

*HnN : Head and Neck

**Supplementary Table 6: Confusion matrix for multi-tissue multi-class model (Testing set)**

| a | b | c | d | e | f | g | h | i | j | k | l | m | n | o | p | q | r | Classified as |
|---|---|---|---|---|---|---|---|---|---|---|---|---|---|---|---|---|---|---|
| 275 | 0 | 0 | 0 | 0 | 0 | 0 | 0 | 0 | 0 | 0 | 0 | 0 | 0 | 0 | 0 | 0 | 0 | a = Blood-tumor |
| 0 | 23 | 0 | 0 | 0 | 0 | 0 | 0 | 0 | 0 | 0 | 0 | 0 | 0 | 0 | 0 | 0 | 0 | b = Blood-normal |
| 0 | 0 | 63 | 0 | 0 | 0 | 0 | 0 | 0 | 0 | 0 | 0 | 0 | 0 | 0 | 0 | 0 | 0 | c = Breast-tumor |
| 0 | 0 | 0 | 22 | 0 | 0 | 0 | 0 | 0 | 0 | 0 | 0 | 0 | 0 | 0 | 0 | 0 | 0 | d = Breast-normal |
| 0 | 0 | 0 | 0 | 45 | 2 | 0 | 0 | 0 | 0 | 0 | 0 | 0 | 0 | 0 | 0 | 0 | 0 | e = Colon-tumor |
| 0 | 0 | 0 | 0 | 3 | 2 | 0 | 0 | 0 | 0 | 0 | 1 | 0 | 0 | 0 | 0 | 0 | 0 | f = Colon-normal |
| 0 | 0 | 0 | 0 | 0 | 0 | 158 | 0 | 0 | 0 | 0 | 0 | 0 | 0 | 0 | 0 | 0 | 0 | g = Gastric-tumor |
| 0 | 0 | 0 | 0 | 0 | 0 | 0 | 8 | 0 | 0 | 0 | 1 | 0 | 0 | 0 | 0 | 0 | 0 | h = Gastric-normal |
| 0 | 0 | 0 | 0 | 0 | 0 | 0 | 0 | 35 | 0 | 0 | 0 | 0 | 0 | 0 | 0 | 0 | 0 | i = HnN-tumor |
| 0 | 1 | 0 | 0 | 0 | 0 | 0 | 0 | 0 | 4 | 0 | 0 | 0 | 0 | 1 | 0 | 0 | 0 | j = HnN-normal |
| 0 | 0 | 0 | 0 | 0 | 0 | 0 | 0 | 0 | 0 | 205 | 0 | 0 | 0 | 0 | 0 | 0 | 0 | k = Lung-tumor |
| 0 | 0 | 0 | 0 | 0 | 0 | 0 | 0 | 0 | 0 | 0 | 66 | 0 | 0 | 0 | 0 | 0 | 0 | l = Lung-normal |
| 0 | 0 | 0 | 0 | 0 | 0 | 0 | 0 | 0 | 0 | 0 | 0 | 17 | 2 | 0 | 0 | 0 | 0 | m = Prostate-tumor |
| 0 | 0 | 0 | 0 | 0 | 0 | 0 | 0 | 0 | 0 | 0 | 0 | 3 | 5 | 1 | 0 | 0 | 0 | n = Prostate-normal |
| 0 | 0 | 0 | 0 | 0 | 0 | 0 | 0 | 0 | 0 | 0 | 0 | 0 | 0 | 29 | 1 | 0 | 0 | o = Thyroid-tumor |
| 0 | 0 | 0 | 0 | 0 | 0 | 0 | 0 | 0 | 0 | 0 | 0 | 0 | 0 | 3 | 79 | 0 | 0 | p = Thyroid-normal |
| 0 | 1 | 0 | 0 | 0 | 0 | 0 | 0 | 0 | 0 | 0 | 0 | 0 | 0 | 0 | 0 | 24 | 1 | q = Tongue-tumor |
| 0 | 0 | 0 | 0 | 0 | 0 | 0 | 0 | 0 | 0 | 0 | 0 | 0 | 0 | 0 | 0 | 7 | 0 | r = Tongue-normal |

*HnN : Head and Neck.

**Supplementary Table 7: Performance of the multi-tissue normal multi-class model for each class**

| Class of samples | # of correctly classified samples (total # of samples in test set) | Precision (%) | Recall (%) | F1-Score |
|---|---|---|---|---|
| Blood | 23 (23) | 100 | 95.83 | 97.87 |
| Breast | 21 (22) | 95.45 | 95.45 | 95.45 |
| Gastric | 7 (8) | 87.5 | 100 | 93.33 |
| Head & Neck | 6 (6) | 100 | 100 | 100 |
| Lung | 66 (66) | 100 | 95.65 | 97.78 |
| Thyroid | 60 (60) | 100 | 100 | 100 |
| Tongue | 1 (1) | 100 | 100 | 100 |

Precision, Recall and F1-Score using the testing data.
*colon and prostate tissue is not shown due to no samples in test set

**Supplementary Table 8: Confusion Matrix for multi-tissue normal multi-class model (training set)**

| a | b | c | d | e | f | g | h | i | classified as |
|---|---|---|---|---|---|---|---|---|---|
| 23 | 0 | 0 | 0 | 0 | 0 | 0 | 0 | 0 | a = Blood |
| 0 | 22 | 0 | 0 | 0 | 0 | 0 | 0 | 0 | b = Breast |
| 0 | 0 | 0 | 0 | 0 | 0 | 0 | 1 | 0 | c = Colon |
| 0 | 0 | 0 | 9 | 0 | 0 | 0 | 0 | 0 | d = Gastric |
| 0 | 0 | 0 | 0 | 5 | 0 | 0 | 1 | 0 | e = Head and Neck |
| 0 | 0 | 0 | 0 | 0 | 66 | 0 | 0 | 0 | f = Lung |
| 0 | 0 | 0 | 0 | 0 | 1 | 1 | 0 | 0 | g = Prostate |
| 0 | 0 | 0 | 0 | 0 | 0 | 0 | 59 | 0 | h = Thyroid |
| 0 | 0 | 0 | 0 | 1 | 0 | 0 | 0 | 0 | i = Tongue |

**Supplementary Table 9: Confusion Matrix for multi-tissue normal multi-class model (testing set)**

| a | b | c | d | e | f | g | h | i | classified as |
|---|---|---|---|---|---|---|---|---|---|
| 23 | 0 | 0 | 0 | 0 | 0 | 0 | 0 | 0 | a = Blood |
| 1 | 21 | 0 | 0 | 0 | 0 | 0 | 0 | 0 | b = Breast |
| 0 | 0 | 0 | 0 | 0 | 1 | 0 | 0 | 0 | c = Colon |
| 0 | 0 | 0 | 7 | 0 | 1 | 0 | 0 | 0 | d = Gastric |
| 0 | 0 | 0 | 0 | 6 | 0 | 0 | 0 | 0 | e = Head and Neck |
| 0 | 0 | 0 | 0 | 0 | 66 | 0 | 0 | 0 | f = Lung |
| 0 | 1 | 0 | 0 | 0 | 1 | 0 | 0 | 0 | g = Prostate |
| 0 | 0 | 0 | 0 | 0 | 0 | 0 | 60 | 0 | h = Thyroid |
| 0 | 0 | 0 | 0 | 0 | 0 | 0 | 0 | 1 | i = Tongue |

## Supplementary Table 10: Breast cancer biomarkers with sensitivity and specificity

| Biomarker | Sensitivity (%) | Specificity (%) | Reference |
|---|---|---|---|
| ATP6AP1 | 91.67 | 30.43 | [1] |
| PDCD6IP | 92.11 | 29.41 | [1] |
| DBT | 97.37 | 29.41 | [1] |
| CSNK1E | 84.21 | 37.25 | [1] |
| FRS3 | 55.26 | 42.00 | [1] |
| RAC3 | 84.21 | 33.33 | [1] |
| HOXD1 | 65.79 | 34.00 | [1] |
| SF3A1 | 83.33 | 36.73 | [1] |
| CTBP1 | 89.47 | 23.53 | [1] |
| C15orf48 | 97.37 | 18.00 | [1] |
| MYOZ2 | 92.11 | 23.53 | [1] |
| EIF3E | 89.47 | 33.33 | [1] |
| BAT4 | 80.00 | 30.61 | [1] |
| ATF3 | 86.84 | 20.00 | [1] |
| BMX | 84.21 | 29.41 | [1] |
| RAB5A | 81.58 | 28.57 | [1] |
| UBAP1 | 92.11 | 26.00 | [1] |
| SOX2 | 94.29 | 18.60 | [1] |
| GPR157 | 100.00 | 13.04 | [1] |
| BDNF | 86.84 | 20.00 | [1] |
| ZMYM6 | 89.47 | 19.61 | [1] |
| SLC33A1 | 86.84 | 26.00 | [1] |
| TRIM32 | 78.95 | 33.33 | [1] |
| ALG10 | 97.37 | 15.69 | [1] |
| TFCP2 | 85.29 | 21.74 | [1] |
| SERPINH1 | 89.47 | 11.76 | [1] |
| SELL | 80.56 | 24.00 | [1] |
| ZNF510 | 88.89 | 20.00 | [1] |
| CA 15.3 | 96.00 | 58.2 | [2] |
| Her-2/neu | 98.00 | 40.00 | [3] |
| NDUFA10 | 68 | 98 | [4] |
| PTPMT1 | 52 | 98 | [4] |
| ITGB1 | 48 | 98 | [4] |
| KIT | 46 | 98 | [4] |
| POLR2L | 43 | 98 | [4] |
| EFNA5 | 41 | 98 | [4] |
| RGS5 | 39 | 98 | [4] |
| STOML2 | 36 | 98 | [4] |
| FAS | 36 | 98 | [4] |
| STAT6 | 33 | 98 | [4] |
| CSRP2 | 31 | 98 | [4] |
| SSBP1 | 31 | 98 | [4] |
| EED | 31 | 98 | [4] |
| CCL28 | 29 | 98 | [4] |
| SRP54 | 29 | 98 | [4] |
| CDKN2A | 27 | 98 | [4] |
| CCL27 | 25 | 98 | [4] |
| fbpA | 25 | 98 | [4] |
| MAP2K1 | 22 | 98 | [4] |
| BRCA1 | 20 | 98 | [4] |
| ENG | 19 | 98 | [4] |
| XPOT | 19 | 98 | [4] |
| DGCR6 | 19 | 98 | [4] |
| PCCA | 19 | 98 | [4] |
| AP3B2 | 19 | 98 | [4] |
| DUSP9 | 18 | 98 | [4] |
| TSPO | 8 | 98 | [4] |
| TRAF4 | 4 | 98 | [4] |
| **Range** | **4-100** | **11.76-98** | |

**Supplementary Table 11: Colon cancer biomarkers with sensitivity and specificity**

| Biomarker | Sensitivity (%) | Specificity (%) | Reference |
|---|---|---|---|
| CCSA-2 | 97.3 | 78.4 | [5] |
| Vimentin | 46 | 90 | [6] |
| IHC | 83 | 90 | [7] |
| MSI | 73 | 90 | [7] |
| gFOBT | 37.5 | 94.5 | [7] |
| iFOBT | 72.5 | 94 | [7] |
| Vimentin | 77.7 | 70 | [7] |
| CEA | 46.5 | 81.5 | [7] |
| CA 19-9 | 35 | 89.5 | [7] |
| TIMP-1 | 55 | 95 | [7] |
| CTCs | 63 | 94 | [7] |
| **Range** | **35–97.3** | **70–95** | |

**Supplementary Table 12: Gastric cancer biomarkers with sensitivity and specificity**

| Biomarker | Sensitivity (%) | Specificity (%) | Reference |
|---|---|---|---|
| Alu DNA sequences | 75 | 63 | [8] |
| Total cfDNA level | 96.67 | 94.11 | [9] |
| *HER2* | 53.9 | 96.7 | [10] |
| *RUNX3* | 95.5 | 62.5 | [11] |
| *KCNA4 + CYP26B1* | 91.3 | 92.1 | [12] |
| *SLC19A3* | 85 | 85 | [13] |
| *FAM5C + MYLK* | 77.6 | 90 | [14] |
| Fibrinogen α-chain | 90.9 | 90.6 | [15] |
| **Range** | **53.9–96.67** | **62.5–96.7** | |

**Supplementary Table 13: Head and neck cancer biomarkers with sensitivity and specificity.** See Supplementary_Table_13

**Supplementary Table 14: Lung cancer biomarkers with sensitivity and specificity**

| Biomarker | Sensitivity (%) | Specificity (%) | Reference |
|---|---|---|---|
| DCC | 35.5 | 100 | [66] |
| APC<br>CDH1<br>MGMT<br>DCC<br>RASSF1A<br>AIM1 | 73 | 75 | [66] |
| carcinoembryonic antigen retinol binding protein<br>alpha 1-antitrypsin<br>squamous cell carcinoma antigen | 89.3 | 84.7 | [67] |
| TP63<br>MYC<br>CEP3<br>CEP6 | 82 | 58 | [68] |
| miRNA-21<br>miRNA-126<br>miRNA-210<br>miRNA-486-5p | 86 | 96 | [69] |
| CCL18 | 91 | 84 | [70] |
| CD98<br>fascin<br>sPIgR4<br>14-3-3 eta | 96 | 77 | [71] |
| Cancer Antigen 125 | 84 | 80 | [72] |
| **Range** | **35.5–96** | **58–100** | |

**Supplementary Table 15: Prostate cancer biomarkers with sensitivity and specificity**

| Biomarker | Sensitivity(%) | Specificity (%) | Reference |
|---|---|---|---|
| PSA | 16 | 95 | [73] |
| TMPRSS2<br>ERG | 37 | 93 | [73] |
| ERG<br>PCA3<br>SPDEF | 92 | 34 | [73] |
| HOXC6<br>DLX1 | 91 | 36 | [73] |
| PSA | 46 | 91 | [74] |
| PCA3 | 66 | 76 | [75] |
| GSTP1<br>APC<br>RASSF1 | 68 | 64 | [76] |
| PHI | 80 | 45 | [77] |
| **Range** | **16–92** | **34–95** | |

## Supplementary Table 16: Tongue cancer biomarkers with sensitivity and specificity

| Biomarker | Sensitivity (%) | Specificity (%) | Reference |
|---|---|---|---|
| HOXA9 | 68 | 100 | [78] |
| NID2 | 71 | 100 | [78] |
| HOXA9+NID2 | 94 | 97 | [78] |
| MMP-9 | 100 | 79 | [79] |
| Carbonyls | 90 | 80 | [79] |
| OGG1 | 77 | 75 | [79] |
| phospho-Src | 77 | 75 | [79] |
| Ki67 | 58 | 67 | [79] |
| Maspin | 100 | 100 | [79] |
| LDH | 79 | 42 | [79] |
| CycD1 | 100 | 100 | [79] |
| **Range** | **58–100** | **42–100** | |

## Supplementary Table 17: Sample distribution of tumor and normal samples by tissue of origin

| Tissue | Tumor | Normal | Total | Train/Test Set |
|---|---|---|---|---|
| Blood | 549 | 46 | 595 | 298 |
| Breast | 127 | 44 | 171 | 86 |
| Colon | 93 | 12 | 105 | 53 |
| Gastric | 316 | 17 | 333 | 167 |
| Head and Neck | 70 | 12 | 82 | 41 |
| Lung | 410 | 132 | 542 | 271 |
| Prostate | 38 | 18 | 56 | 28 |
| Thyroid | 60 | 164 | 224 | 112 |
| Tongue | 53 | 14 | 67 | 34 |
| **Total** | **1716** | **459** | **2175** | **1088** |

## REFERENCES


1. Anderson KS, Sibani S, Wallstrom G, Qiu J, Mendoza E a, Raphael J, Hainsworth E, Montor WR, Wong J, Park JG, Lokko N, Logvinenko T, Ramachandran N, et al. Protein microarray signature of autoantibody biomarkers for the early detection of breast cancer. J Proteome Res. 2011; 10:85–96. https://doi.org/10.1021/ pr100686b.
2. Ciambellotti E, Coda C, Lanza E. Determination++ of CA 15-3 in the control of primary and metastatic breast carcinoma. Minerva Med. 1993; 84:107–12. http://www.ncbi.nlm.nih.gov/pubmed/8492961.
3. Cook GB, Neaman IE, Goldblatt JL, Cambetas DR, Hussain M, Lüftner D, Yeung KK, Chan DW, Schwartz MK, Allard WJ. Clinical utility of serum HER-2/neu testing on the bayer immuno 1?? automated system in breast cancer. Anticancer Res. 2001; 21:1465–70. http://www.ncbi.nlm.nih.gov/pubmed/11396233.
4. Li CI, Mirus JE, Zhang Y, Ramirez AB, Ladd JJ, Prentice RL, McIntosh MW, Hanash SM, Lampe PD. Discovery and preliminary confirmation of novel early detection biomarkers for triple-negative breast cancer using preclinical plasma samples from the Women's Health Initiative observational study. Breast Cancer Res Treat. 2012; 135:611–8. https://doi.org/10.1007/ s10549-012-2204-4.
5. Leman ES, Schoen RE, Magheli A, Sokoll LJ, Chan DW, Getzenberg RH. Evaluation of Colon Cancer–Specific Antigen 2 as a Potential Serum Marker for Colorectal Cancer. Clin Cancer Res. 2008;14:1349-54.
6. Chen WD, Han ZJ, Skoletsky J, Olson J, Sah J, Myeroff L, Platzer P, Lu S, Dawson D, Willis J, Pretlow TP, Lutterbaugh J, Kasturi L, et al. Detection in fecal DNA of colon cancer-specific methylation of the nonexpressed vimentin gene. J Natl Cancer Inst. 2005; 97:1124–32. https://doi.org/10.1093/jnci/dji204.
7. Gonzalez-Pons M, Cruz-Correa M. Colorectal Cancer Biomarkers: Where Are We Now? Biomed Res Int. 2015; 2015: 149014. https://doi. org/10.1155/2015/149014.
8. Park J-L, Kim HJ, Choi BY, Lee H-C, Jang H-R, Song KS, Kim YS. Quantitative analysis of cell-free DNA in the plasma of gastric cancer patients. Oncol Lett. 2012; 3:921– 6. https://doi.org/10.3892/ol.2012.592.
9. Kim K, Shin DG, Park MK, Baik SH, Kim TH, Kim S, Lee S. Circulating cell-free DNA as a promising biomarker in patients with gastric cancer: diagnostic validity and significant reduction of cfDNA after surgical resection. Ann Surg Treat Res. 2014; 86:136–42. https://doi.org/10.4174/astr.2014.86.3.136.
10. Shoda K, Masuda K, Ichikawa D, Arita T, Miyakami Y, Watanabe M, Konishi H, Imoto I, Otsuji E. HER2 amplification detected in the circulating DNA of patients with gastric cancer: a retrospective pilot study. Gastric Cancer. 2015; 18:698–710. https://doi.org/10.1007/s10120- 014-0432-5.
11. Sakakura C, Hamada T, Miyagawa K, Nishio M, Miyashita A, Nagata H, Ida H, Yazumi S, Otsuji E, Chiba T, Ito K, Ito Y. Quantitative analysis of tumor-derived methylated RUNX3 sequences in the serum of gastric cancer patients. Anticancer Res. 2009; 29:2619–25. http:// www.ncbi.nlm.nih.gov/pubmed/19596937.
12. Zheng Y, Chen L, Li J, Yu B, Su L, Chen X, Yu Y, Yan M, Liu B, Zhu Z. Hypermethylated DNA as potential biomarkers for gastric cancer diagnosis. Clin Biochem. 2011; 44:1405–11. https://doi.org/10.1016/j.clinbiochem.2011.09.006.
13. Ng EKO, Leung CPH, Shin VY, Wong CLP, Ma ESK, Jin HC, Chu KM, Kwong A. Quantitative analysis and diagnostic significance of methylated SLC19A3 DNA in the plasma of breast and gastric cancer patients. PLoS One. 2011; 6: e22233. https://doi.org/10.1371/ journal.pone.0022233.
14. Chen L, Su L, Li J, Zheng Y, Yu B, Yu Y, Yan M, Gu Q, Zhu Z, Liu B. Hypermethylated FAM5C and MYLK in serum as diagnosis and pre-warning markers for gastric cancer. Dis Markers. 2012; 32:195–202. https://doi.org/10.3233/DMA-2011-0877.
15. Liu W, Liu B, Cai Q, Li J, Chen X, Zhu Z. Proteomic identification of serum biomarkers for gastric cancer using multi-dimensional liquid chromatography and 2D differential gel electrophoresis. Clin Chim Acta. 2012; 413:1098–106. https://doi.org/10.1016/j.cca.2012.03.003.
16. Aggarwal S, Devaraja K, Sharma SC, Das SN. Expression of vascular endothelial growth factor (VEGF) in patients with oral squamous cell carcinoma and its clinical significance. Clin Chim Acta. 2014; 436:35–40. https://doi. org/10.1016/j.cca.2014.04.027.
17. Aggarwal S, Sharma SC, Das SN. Galectin-1 and galectin-3: Plausible tumour markers for oral squamous cell carcinoma and suitable targets for screening high-risk population. Clin Chim Acta. 2015; 442:13–21. https://doi.org/10.1016/j.cca.2014.12.038.
18. Al D, Álvarez C, Blanco I. [Diagnostic value of E-cadherin, MMP-9, activated MMP-13 and anti-p53 antibodies in squamous cell carcinomas of head and neck]. Med Clin (Barc). 2007; 129:761–5. https:// doi.org/10.1157/13113764. [Article in Spanish].
19. Ayude D, Gacio G, Paez de la Cadena M, Pallas E, Martinez-Zorzano VS, de Carlos A, Rodriguez-Berrocal FJ. Combined use of established and novel tumour markers in the diagnosis of head and neck squamous cell carcinoma. Oncol Rep. 2003; 10:1345–50. http://www. ncbi.nlm.nih.gov/pubmed/12883705.
20. Bhatavdekar JM, Patel DD, Vora HH, Shah NG, Karelia NH, Ghosh N, Balar DB. Prolactin: Its role in advanced tongue cancer. J Surg Oncol. 1994; 57:115–20. https://doi.org/10.1002/jso.2930570209.
21. Ceruse P, Rabilloud M, Charrie A, Dubreuil C, Disant F. Study of {Cyfra 21-1}, a tumor marker, in head and neck squamous cell carcinoma. Ann Otol Rhinol Laryngol. 2005; 114:768–76. https://doi.org/10.1177/000348940511401006.

22. Cheng A-J, Chen L-C, Chien K-Y, Chen Y-J, Chang JTC, Wang H-M, Liao C-T, Chen I-H. Oral Cancer Plasma Tumor Marker Identified with Bead-Based Affinity Fractionated Proteomic Technology. Clin Chem. 2005;51:2236-44.

23. Chen CH, Chuang HC, Huang CC, Fang FM, Huang HY, Tsai HT, Su LJ, Shiu LY, Leu S, Chien CY. Overexpression of rap-1A indicates a poor prognosis for oral cavity squamous cell carcinoma and promotes tumor cell invasion via aurora-A modulation. Am J Pathol. 2013; 182:516–28. https://doi.org/10.1016/j.ajpath.2012.10.023.

24. Choudhury B, Srivastava S, Choudhury HH, Purkayastha A, Duttagupta S, Ghosh SK. Arginase and C-reactive protein as potential serum-based biomarker of head and neck squamous cell carcinoma patients of north east India. Tumour Biol. 2014; 35:6739–48. https://doi.org/10.1007/s13277-014- 1851-y.

25. Deng YF, Chen P, Lin YZ, Le JZ, Wu XL, Yu MQ, Zhuang PY, Gao MH. Analytical and clinical evaluation of CYFRA 21-1 by electrochemiluminescent immunoassay in head and neck squamous cell carcinoma. J Laryngol Otol. 2003; 117:190–4. https://doi.org/http://dx.doi. org/10.1258/002221503321192485.

26. Doweck I, Barak M, Greenberg E, Uri N, Kellner J, Lurie M. Cyfra 21-1. A new potential tumor marker for squamous cell carcinoma of head and neck. Arch Otolaryngol Head Neck Surg. 1995; 121:177–81. https://doi.org/10.1001/archotol.1995.01890020039009.

27. Ervens J, Fuchs H, Niemann VT, Hoffmeister B. Pyruvate kinase isoenzyme M2 is not of diagnostic relevance as a marker for oral cancer. J Cranio-Maxillofacial Surg. 2008; 36:89–94. https://doi.org/10.1016/j.jcms.2007.08.006.

28. Feng XY, Li JH, Li JZ, Li H, Han ZX, Xing RD. Serum SCCA, Cyfra 21-1, EGFR and cyclin D1 levels in patients with oral squamous cell carcinoma. Int J Biol Markers. 2010; 25:93–8. http://www.ncbi.nlm.nih. gov/pubmed/20586028.

29. Goumas PD, Mastronikolis NS, Mastorakou AN, Vassilakos PJ, Nikiforidis GC. Evaluation of TATI and CYFRA 21-1 in patients with head and neck squamous cell carcinoma. ORL J Otorhinolaryngol. 1997; 59:106–114. https://doi.org/10.1159/000276919.

30. Charushila YK, Raghavendra VK, Adinath NS, Kumbar KM, Kadam DP. Biochemical markers in oral cancer. Biomed Res. 2011; 22:76–80.

31. Kandiloros D, Eleftheriadou A, Chalastras T, Kyriou L, Yiotakis I, Ferekidis E. Prospective study of a panel of tumor markers as prognostic factors in patients with squamous cell carcinoma of head and neck. Med Oncol. 2006; 23:463–70. https://doi.org/10.1385/ MO:23:4:463.

32. Kimoto A, Nishiumi S, Kobayashi T, Terashima Y, Suzuki H, Takeuchi J, Azuma T, Komori T, Yoshida M. A novel gas chromatography mass spectrometry-based serum screening method for oral squamous cell carcinoma . Head Neck Oncol. 2013; 5.

33. Krecicki T, Siewiński M. Serum cathepsin B-like activity as a potential marker of laryngeal carcinoma. Eur Arch Otorhinolaryngol. 1992; 249:293–5. http:// www.ncbi.nlm.nih.gov/pubmed/1524813.

34. Krishnan R, Thayalan DK, Padmanaban R, Ramadas R, Annasamy RK, Anandan N. Association of serum and salivary tumor necrosis factor-α with histological grading in oral cancer and its role in differentiating premalignant and malignant oral disease. Asian Pacific J Cancer Prev. 2014; 15:7141– 8. https://doi.org/10.7314/APJCP.2014.15.17.7141.

35. Kurokawa H, Yamashita Y, Tokudome S, Kajiyama M. Combination assay for tumor markers in oral squamous cell carcinoma. J Oral Maxillofac Surg. 1997; 55:964–6. https://doi.org/10.1016/S0278-2391(97)90071-9.

36. Lee J, Jeong S, Lee CR, Ku CR, Kang S-W, Jeong JJ, Nam K-H, Shin DY, Chung WY, Lee EJ, Jo YS. GLI1 Transcription Factor Affects Tumor Aggressiveness in Patients With Papillary Thyroid Cancers. Medicine (Baltimore). 2015; 94: e998. https://doi.org/10.1097/MD.0000000000000998.

37. Li X, Qiao Z, Long X, Wei J, Cheng Y. Serum concentration of AMDL DR-70 for the diagnosis and prognosis of carcinoma of the tongue. Br J Oral Maxillofac Surg. 2005; 43:513–5. https://doi.org/10.1016/j.bjoms.2004.11.020.

38. Liu CJ, Kao SY, Tu HF, Tsai MM, Chang KW, Lin SC. Increase of microRNA miR-31 level in plasma could be a potential marker of oral cancer. Oral Dis. 2010; 16:360–4. https://doi.org/10.1111/j.1601-0825.2009.01646.x.

39. Liu Y, Zhou ZT, He QB, Jiang WW. DAPK promoter hypermethylation in tissues and body fluids of oral precancer patients. Med Oncol. 2012; 29:729– 33. https://doi.org/10.1007/s12032-011-9953-5.

40. Liu L, Liu B, Zhu L-L, Li Y. CYFRA21-1 as a serum tumor marker for follow-up patients with squamous cell lung carcinoma and oropharynx squamous cell carcinoma. Biomark Med. 2013; 7:591–9. https://doi.org/10.2217/bmm.13.55.

41. Lu YC, Chang JTC, Huang YC, Huang CC, Chen WH, Lee LY, Huang BS, Chen YJ, Li HF, Cheng AJ. Combined determination of circulating miR-196a and miR-196b levels produces high sensitivity and specificity for early detection of oral cancer. Clin Biochem. 2015; 48:115–21. https://doi.org/10.1016/j.clinbiochem.2014.11.020.

42. Maass JD, Niemann AM, Lippert BM, Gottschlich S, Folz BJ, Werner JA. [Determination of Serum Soluble Fragments of Cytokeratin 8 and 18 in Patients with Squamous Cell Carcinomas of the Head and Neck.] Laryngo-Rhino-Otologie. 1997; 76:554–8. https://doi.org/10.1055/s-2007-997478. [Article in German].

43. Marcos CÁ, Martínez DAK, de los Toyos JR, Domínguez Iglesias F, Hermsen M, Guervós MA, Pendás JLL. The usefulness of new serum tumor markers in head and neck squamous cell carcinoma. Otolaryngol Head Neck Surg. 2009; 140:375–80. https://doi.org/10.1016/j.otohns.2008.12.026.

44. Mevio E, Benazzo M, Galioto P, Spriano P, Pizzala R. Use of serum markers in the diagnosis and management of laryngeal

cancer. Clin Otolaryngol Allied Sci. 1991;16:90–2. https://doi.org/10.1111/j.1365-2273.1991. tb01950.x.

45. Moergel M, Kämmerer PW, Schnurr K, Klein MO, AlNawas B. Spin electron paramagnetic resonance of albumin for diagnosis of oral squamous cell carcinoma (OSCC). Clin Oral Invest. 2012; 16:1529–33. https:// doi.org/10.1007/s00784-011-0655-3.

46. Murase R, Abe Y, Takeuchi T, Nabeta M, Imai Y, Kamei Y, Kagawa-Miki L, Ueda N, Sumida T, Hamakawa H, Kito K. Serum autoantibody to sideroflexin 3 as a novel tumor marker for oral squamous cell carcinoma. Proteomics Clin Appl. 2008; 2:517–27. https://doi. org/10.1002/prca.200780123.

47. Nagler RM, Barak M, Peled M, Ben-Aryeh H, Filatov M, Laufer D. Early diagnosis and treatment monitoring roles of tumor markers Cyfra 21-1 and TPS in oral squamous cell carcinoma. Cancer. 1999; 85:1018–25.

48. Nayak S, Goel MM, Chandra S, Bhatia V, Mehrotra D, Kumar S, Makker A, Rath SK, Agarwal SP. VEGF-A immunohistochemical and mRNA expression in tissues and its serum levels in potentially malignant oral lesions and oral squamous cell carcinomas. Oral Oncol. 2012; 48:233– 9. https://doi.org/10.1016/j.oraloncology.2011.10.003.

49. Palermo F, Carniato A, Fede A, Boccaletto F, Marchiori C. Serum SCC-Ag in head and neck squamous cell carcinoma. Int J Biol Markers. 1990;5:118-20. http://www. ncbi.nlm.nih.gov/pubmed/2286775.

50. Qiu G, Li Y, Liu Z, Wang M, Ge J, Bai X. Clinical value of serum HMGB1 in diagnosis and prognosis of laryngeal squamous cell carcinoma. Med Oncol. 2014;31: 316. https://doi.org/10.1007/s12032- 014-0316-x.

51. Rajkumar K, Ramya R, Nandhini G, Rajashree P, Ramesh Kumar A, Nirmala Anandan S. Salivary and serum level of CYFRA 21-1 in oral precancer and oral squamous cell carcinoma. Oral Dis. 2015; 21:90–6. https://doi.org/10.1111/odi.12216.

52. Rasool M, Khan SR, Malik A, Khan KM, Zahid S, Manan A, Qazi MH, Naseer MI. Comparative Studies of Salivary and Blood Sialic Acid, Lipid Peroxidation and Antioxidative Status in Oral Squamous Cell Carcinoma (OSCC). Pakistan J Med Sci. 2014; 30:466–71. https://doi.org/10.12669/pjms.303.4985.

53. Rathan Shetty SK, Kumar Bhandary S, Kali A. Significance of serum L-fucose glycoprotein as cancer biomarker in head and neck malignancies without distant metastasis. J Clin Diagnostic Res. 2013; 7:2818–20. https://doi.org/10.7860/JCDR/2013/6681.3765.

54. Ries J, Vairaktaris E, Agaimy A, Kintopp R, Baran C, Neukam FW, Nkenke E. miR-186, miR-3651 and miR-494: Potential biomarkers for oral squamous cell carcinoma extracted from whole blood. Oncol Rep. 2014; 31:1429–36. https://doi.org/10.3892/or.2014.2983.

55. Ropka ME, Goodwin WJ, Levine PA, Sasaki CT, Kirchner JC, Cantrell RW. Effective head and neck tumor markers. The continuing quest. Arch Otolaryngol Head Neck Surg. 1991; 117:1011–4. http://www.ncbi.nlm.nih. gov/pubmed/1910716.

56. Saussez S, Glinoer D, Chantrain G, Pattou F, Carnaille B, André S, Gabius H, Laurent G. Serum galectin-1 and galectin-3 levels in benign and malignant nodular thyroid disease. Thyroid. 2008; 18:705–12. https://doi.org/10.1089/thy.2007.0361.

57. Schiegnitz E, Kämmerer PW, Koch FP, Krüger M, Berres M, Al-Nawas B. GDF 15 as an anti-apoptotic, diagnostic and prognostic marker in oral squamous cell carcinoma. Oral Oncol. 2012; 48:608–14. https://doi.org/10.1016/j.oraloncology.2012.01.020.

58. Chaudhary AK, Pandya S, Mehrotra R, Singh M, Singh M. Role of functional polymorphism of matrix metalloproteinase-2 (-1306 C/T and -168 G/T) and MMP-9 (-1562 C/T) promoter in oral submucous fibrosis and head and neck squamous cell carcinoma in an Indian population. Biomarkers. 2011; 16:577–86. https://doi.org/10.3109/135475 0X.2011.609602.

59. St John MA, Li Y, Zhou X, Denny P, Ho CM, Montemagno C, Shi W, Qi F, Wu B, Sinha U, Jordan R, Wolinsky L, Park NH, et al. Interleukin 6 and interleukin 8 as potential biomarkers for oral cavity and oropharyngeal squamous cell carcinoma. Arch Otolaryngol Head Neck Surg. 2004; 130:929–35. https://doi.org/10.1001/archotol.130.8.929.

60. Vajaria BN, Patel KR, Begum R, Shah FD, Patel JB, Shukla SN, Patel PS. Evaluation of serum and salivary total sialic acid and ??-l-fucosidase in patients with oral precancerous conditions and oral cancer. Oral Surg Oral Med Oral Pathol Oral Radiol. 2013; 115:764–71. https://doi.org/10.1016/j.oooo.2013.01.004.

61. Wang J, Zhou Y, Lu J, Sun Y, Xiao H, Liu M, Tian L. Combined detection of serum exosomal miR-21 and HOTAIR as diagnostic and prognostic biomarkers for laryngeal squamous cell carcinoma. Med Oncol. 2014; 31: 148. https://doi.org/10.1007/s12032-014- 0148-8.

62. Wollenberg B, Jan V, Schmit UM, Hofmann K, Stieber P, Fateh-Moghadam A. CYFRA 21-1 is not superior to SCC antigen and CEA in head and neck squamous cell cancer. Anticancer Res. 1996; 16:3117–24.

63. Wollenberg B, Jan N, Sautier W, Hofmann K, Schmitt UM, Stieber P. Serum levels of intercellular adhesion molecule-1 in squamous cell carcinoma of the head and neck. Tumour Biol. 1997; 18:88–94.

64. Zhang W, Zhang Q, Zhang M, Zhang Y, Li F, Lei P. Network analysis in the identification of special mechanisms between small cell lung cancer and non-small cell lung cancer. Thorac Cancer. 2014; 5:556–64. https:// doi.org/10.1111/1759-7714.12134.

65. Zhong L ping, Zhang C ping, Zheng J wei, Li J, Chen W tao, Zhang Z yuan. Increased Cyfra 21-1 concentration in saliva from primary oral squamous cell carcinoma patients. Arch Oral Biol. 2007; 52:1079–87. https://doi.org/10.1016/j.archoralbio.2007.05.005.

66. Begum S, Brait M, Dasgupta S, Ostrow KL, Zahurak M, Carvalho AL, Califano JA, Goodman SN, Westra WH, Hoque MO, Sidransky D. An epigenetic marker panel for detection of lung cancer using cell-free serum DNA. Clin Cancer Res. 2011; 17:4494–503. https://doi. org/10.1158/1078-0432. CCR-10-3436.

67. Patz EF, Campa MJ, Gottlin EB, Kusmartseva I, Xiang RG, Herndon JE. Panel of serum biomarkers for the diagnosis of lung cancer. J Clin Oncol. 2007; 25:5578–83. https://doi. org/10.1200/JCO.2007.13.5392.

68. Massion PP, Zou Y, Uner H, Kiatsimkul P, Wolf HJ, Baron AE, Byers T, Jonsson S, Lam S, Hirsch FR, Miller YE, Franklin WA, Varella-Garcia M. Recurrent genomic gains in preinvasive lesions as a biomarker of risk for lung cancer. PLoS One. 2009; 4: e5611. https://doi. org/10.1371/journal. pone.0005611.

69. Zhong L, Hidalgo GE, Stromberg AJ, Khattar NH, Jett JR, Hirschowitz EA. Using protein microarray as a diagnostic assay for non-small cell lung cancer. Am J Respir Crit Care Med. 2005; 172:1308–14. https://doi.org/10.1164/ rccm.200505-830OC.

70. Ostroff RM, Bigbee WL, Franklin W, Gold L, Mehan M, Miller YE, Pass HI, Rom WN, Siegfried JM, Stewart A, Walker JJ, Weissfeld JL, Williams S, et al. Unlocking biomarker discovery: Large scale application of aptamer proteomic technology for early detection of lung cancer. PLoS One. 2010; 5: e15003. https://doi. org/10.1371/journal. pone.0015003.

71. Xiao T. An Approach to Studying Lung Cancer-related Proteins in Human Blood. Mol Cell Proteomics. 2005; 4:1480–6. https://doi.org/10.1074/mcp.M500055-MCP200.

72. Dabrowska M, Grubek-Jaworska H, Domagała-Kulawik J, Bartoszewicz Z, Kondracka A, Krenke R, Nejman P, Chazan R. [Diagnostic usefulness of selected tumor markers (CA125, CEA, CYFRA 21-1) in bronchoalveolar lavage fluid in patients with non-small cell lung cancer.] Pol Arch Med Wewn. 2004; 111:659–65. http://www.ncbi. nlm.nih.gov/ pubmed/15508787 [Article in Polish].

73. McGrath S, Christidis D, Perera M, Hong SK, Manning T, Vela I, Lawrentschuk N. Prostate Cancer Biomarkers: Are we hitting the mark? Prostate Int. 2016; 4:130–5. https://doi. org/10.1016/j.prnil.2016.07.002.

74. Gann PH, Hennekens CH, Stampfer MJ. A prospective evaluation of plasma prostate-specific antigen for detection of prostatic cancer. JAMA. 1995; 273:289–94. https://doi. org/10.1001/jama.273.4.289.

75. Wallner LP, Frencher SK, Hsu J-WY, Chao CR, Nichol MB, Loo RK, Jacobsen SJ. Changes in serum prostate-specific antigen levels and the identification of prostate cancer in a large managed care population. BJU Int. 2013; 111:1245– 52. https://doi.org/10.1111/j.1464-410X.2012.11651.x.

76. Van Neste L, Herman JG, Otto G, Bigley JW, Epstein JI, Van Criekinge W. The Epigenetic promise for prostate cancer diagnosis. Prostate. 2012; 72:1248–61. https://doi. org/10.1002/pros.22459.

77. Van Gils MPMQ, Hessels D, Van Hooij O, Jannink SA, Peelen WP, Hanssen SLJ, Witjes JA, Cornel EB, Karthaus HFM, Smits GAHJ, Dijkman GA, Mulders PFA, Schalken JA. The time-resolved fluorescence-based PCA3 test on urinary sediments after digital rectal examination; a Dutch multicenter validation of the diagnostic performance. Clin Cancer Res. 2007; 13:939–43. https://doi.org/10.1158/1078-0432.CCR-06-2679.

78. Guerrero-Preston R, Soudry E, Acero J, Orera M, MorenoLópez L, Macía-Colón G, Jaffe A, Berdasco M, Ili-Gangas C, Brebi-Mieville P, Fu Y, Engstrom C, Irizarry RA, et al. NID2 and HOXA9 promoter hypermethylation as biomarkers for prevention and early detection in oral cavity squamous cell carcinoma tissues and saliva. Cancer Prev Res. 2011; 4:1061–72. https://doi.org/10.1158/1940-6207. CAPR-11-0006.

79. Shpitzer T, Hamzany Y, Bahar G, Feinmesser R, Savulescu D, Borovoi I, Gavish M, Nagler RM. Salivary analysis of oral cancer biomarkers. Br J Cancer. 2009; 101:1194–8. https:// doi.org/10.1038/sj.bjc.6605290.
